# Economic evaluations in medical technological innovations a mapping review of methodologies

**DOI:** 10.1186/s12962-024-00529-0

**Published:** 2024-03-19

**Authors:** C. van Lieshout, G. W. J. Frederix, L. Schoonhoven

**Affiliations:** 1 Department of Epidemiology and Health Economics, Julius Center for Health Sciences and Primary Care, University Medical Center Utrecht, Utrecht University, Utrecht, The Netherlands; 2 The Healthcare Innovation Center (THINC), Julius Center for Health Sciences and Primary Care, University Medical Center Utrecht, Utrecht University, Utrecht, The Netherlands; 3 Department of General Practice and Nursing Science, Julius Center for Health Sciences and Primary Care, University Medical Center Utrecht, Utrecht University, Utrecht, The Netherlands

## Abstract

**Rationale:**

Economic evaluations play an important role in the development and implementation of healthcare innovations. For pharmaceutical products, the methodologies used are laid down in guidelines, whereas for medical technologies the guidelines are not as strenuous. The aim of this review was therefore to analyze what types of methodologies are used in economic evaluations of medical technologies.

**Methods:**

We performed a mapping review to identify economic evaluations for medical technologies. We decided to limit our search to one year (2022) and included cost utility and cost effectiveness analyses in which health technologies were evaluated. For each included study we identified the main methodological characteristics.

**Results:**

A total of 364 papers were included in the analysis, 268 (74%) contained cost-utility analyses and 91 (25%) cost-effectiveness analyses. A model was used in 236 (64%) analyses, 117 analyses were trial based evaluations. Probabilistic sensitivity analyses and/or bootstrapping was performed in 266 (73%) analyses. Deterministic sensitivity analyses were used in 306 (84%). Time horizon and perspective were underreported in 15–25% of the included studies.

**Conclusions:**

This review shows the wide range of methodologies used in economic evaluations as well as the extent and rigor in which these methodologies are used. Many of the included papers did no use or did not sufficiently report the use of appropriate standard methods. This may lead to research waste, a delay in successful implementation of valuable innovations and in the end may delay improvement patient outcomes.

**Supplementary Information:**

The online version contains supplementary material available at 10.1186/s12962-024-00529-0.

## Introduction

Economic evaluations in which costs and effects of standard care and healthcare innovations, e.g. technologies, pharmaceuticals, or organizational changes, are compared are essential elements in the decision-making process of adopting healthcare innovations in many countries. Traditionally these economic evaluations are performed after extensive clinical trials, thus in later stages of product development. In pharmaceuticals, outcomes of economic evaluations are directly used to inform adoption and reimbursement procedures thereby being part of a formal evaluation process [1, 2]. High quality models and/or high-quality data are of utmost importance as the results are directly used to inform these adoption and reimbursement decisions. Therefore, procedures and guidelines are very well established. Given the protocolized nature of the economic evaluation process of pharmaceuticals and often similar timing, data availability is often guaranteed. In addition, the aforementioned guidelines stress that researchers should adhere to various basic development, analysis and reporting standards to ensure high quality, transparency and even reproducibility of health economic evaluations [3, 4].

Over the last decades the use of economic evaluation techniques and corresponding guidelines have also gained attention for medical technologies, which have become an important driver of health care improvement [5]. The standard methodologies used in these economic evaluations are often derived from established practices in the evaluation of pharmaceutical innovations. While these standard methodologies such as perspective, time horizon and sensitivity analysis are essential for ensuring validity and trustworthiness of outcomes, it is the question whether these standard methodologies are useful in the evaluation of non-pharmaceutical medical technologies such as genetic tests, robotics, e-health applications or diagnostic imaging techniques. Another question is whether the methodologies used in economic evaluations of medical technologies are adequately reported as their use is less formally embedded in guidelines for performing economic evaluations, even though reporting checklists such as the Consolidated Health Economic Evaluation Reporting Standards (CHEERS) checklist do require the detailed reporting of these analysis characteristics.

Apart from the need for high-quality models and proper reporting of the economic evaluations of innovations in late stages, we see a trend towards evaluating innovations in earlier stages of development. As research and development of these new medical technologies and devices may take several years, prototypes and early product versions are made which can be used for technological and clinical validation [1, 6, 7]. During the different stages of the technology developmental process, economic evaluations can be performed. These economic evaluations give insight into room for improvement of the innovation but also outline the possible health economic effects of the innovations at an early stage [1, 6, 8]. The use and reporting of methodologies are therefore essential in early analyses due to a higher likelihood of uncertainty as head-to-head comparison data is often lacking. The question is whether all methodologies used in later development stage analyses are and should also be used in analyses of early development stage innovations.

Reviews of health economic analyses often focus on one disease area, one innovation or one patient group. The disadvantage of such an approach is that you often cannot draw conclusions from a large number of studies and or identify forthcoming trends in time. To create an extensive overview and to establish insights into current methodological trends we wanted this review to focus on the methodologies used in published health economic evaluations irrespective of innovation, patient group and or disease.

The primary aim of this study is therefore to establish insight into what types of standard methodologies are used for the economic evaluation of medical technologies. The secondary aim is to distinguish whether differences in standard methods used are present for different stages of technology development.

## Methods

We performed a mapping review of scientific literature to identify economic evaluations in different stages of the product development cycle for medical technologies. In mapping reviews a range of literature within a specified timeframe, in a specified topic area is examined [9]. As this is a mapping review no critical appraisal of included papers was performed [10]. We decided to limit our search to one year (2022) as we aimed to get an overview of the methodologies used and therefore a recent snapshot of published literature was taken.

In January 2024 we searched the PubMed database according to a predefined search, see appendix I. The main subjects of the search related to cost-effectiveness, cost-utility, and economic evaluations. The inclusion and exclusion criteria used can be found in Table 1.

**Table 1:** Inclusion and exclusion criteria

Inclusion criteria	Exclusion criteria
Cost-Effectiveness Analysis *(Head-to-head comparison of alternatives)*	Pharmaceuticals
Cost-Utility Analysis *(Head-to-head comparison of alternatives)*	Screening programs
Medical/Health Technology	Reviews
	No Evaluation of an Innovation
	Non original research papers
	Formal HTA reports
	Economic Evaluation not the primary aim of paper.
	Non-Human care
	No Abstract or full text Available
	Non-English or Non-Dutch

All records retrieved with the search were first uploaded to Endnote to remove duplicates. Next, we uploaded the records to Rayyan screening software. First, the records were screened on title and abstract by CvL, followed by a second closer screening on abstract by CvL as well. The decisions were checked by GF using a 5% sample of records without showing previous decisions by CvL. No differences in inclusion decisions between CvL and GF were found.

### Data extraction and analysis

Relevant characteristics of the studies are described in Table 2. Apart from general paper information, data on the innovation and extent of the standard methodologies used was identified. To address the secondary aim of this paper the stage of development of the innovation is determined as early or late analysis. When the development stage was not explicitly stated it was based on the type of data available on the clinical effectiveness of the innovation. A lack of data, or only data from pilot studies resulted in an “early” classification and when data from extensive clinical studies, registries or routine care is used the analysis was deemed to be an analysis of an innovation in a “late” stage of development. When the intended use of the analysis was to inform reimbursement or implementation the innovation was considered “late” stage. When the intended use was to inform development or further research the innovation or analysis was considered to be “early” stage. This division was made in accordance with the stages defined by IJzerman and Steuten [5].

**Table 2:** Characteristics of studies identified from manuscripts

Group	Parameters
Paper Information	Authors
	Year
	Journal
	Type of Journal (Health Economics/Clinical/Other)
	Title
	ISSN/DOI
Innovation characteristics	Country of Analysis (affiliation 1st Author if not specified elsewhere)
	Specialty
	Main type of Innovation
	Type of Innovation
	Development Phase
	Research Phase
Standard Methodologies Used	Type of Analysis (e.g., CEA/CUA)
	Model Type
	Main Data Source Costs
	Main Data Source Effects
	Effectiveness Measure
	Analysis
	Bootstrapping/PSA
	Sensitivity Analyses
	Value of Information
	Perspective
	Time horizon
	Intended Decision maker
	CHEERS checklist declared

CEA: Cost Effectiveness Analysis, CUA: Cost Utility Analysis, PSA: Probabilistic Sensitivity Analysis, CHEERS: Consolidated Health Economic Evaluation Reporting Standards

## Results

**Fig. 1:**
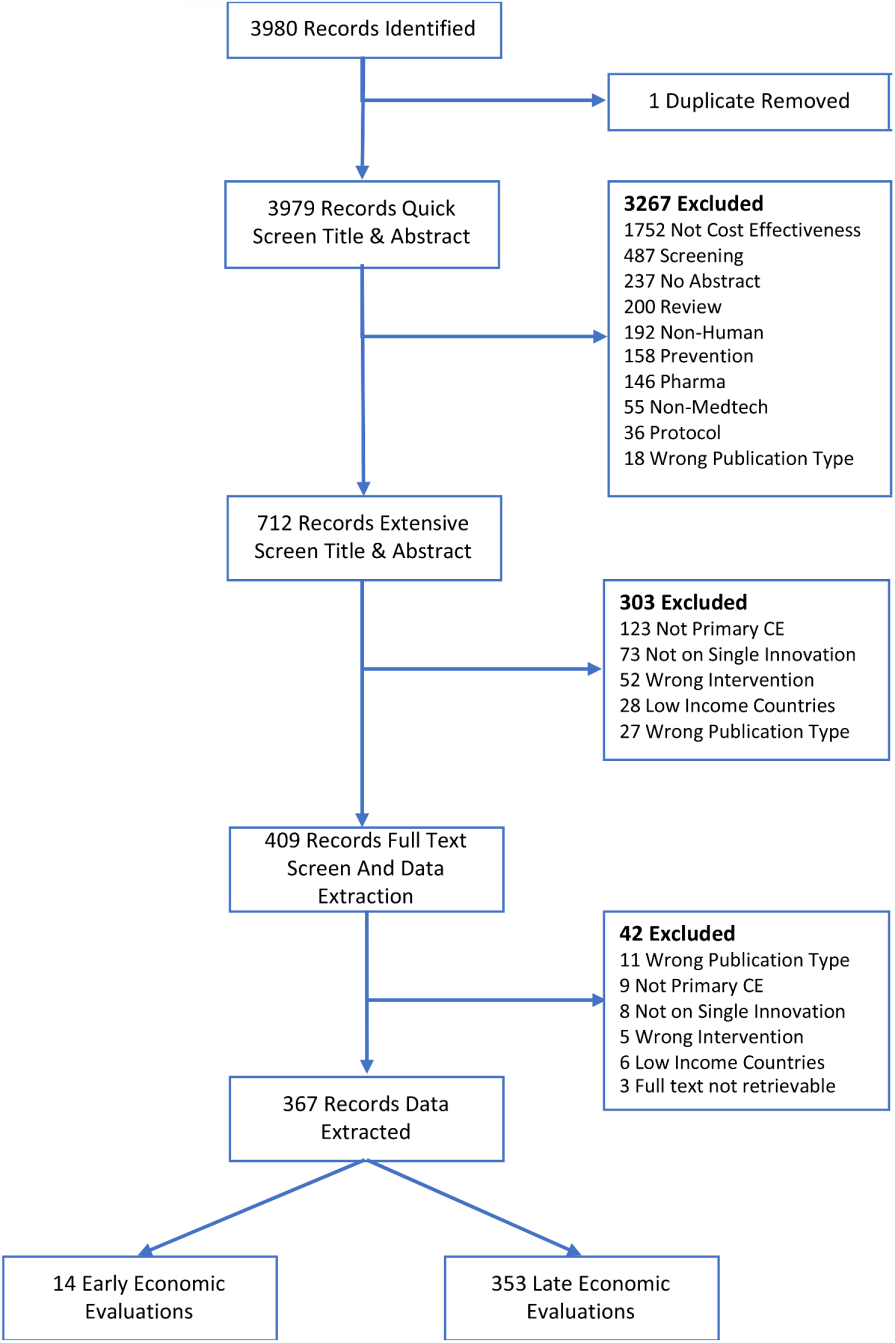
Flowchart of search and inclusion of papers

Figure 1 shows the flowchart of the screening process. The search resulted in a total of 5,079 records, after 20 duplicates were removed the remaining 5,059 were screened on title and abstract. This resulted in the exclusion of 4,612 records, mainly because the analysis did not concern cost-utility or cost-effectiveness analyses (*n =* 1,380*)* followed by papers concerning reviews (*n =* 1,020) and records concerning innovations other than health technologies (*n =* 865). In the end, 447 records were screened on full text, 83 were excluded on full text. Reasons for exclusion were cost analyses (*n =* 48), official HTA reports (*n* = 19), not a primary cost-effectiveness or cost-utility analysis (*n =* 5), full text not retrievable (*n =* 4), on innovation not of interest (non-medtech) (*n =* 4), protocol, review, or wrong language, all (*n* = 1). Data was extracted from the remaining 364 papers, supplemental references S-1 to S-364.

Table 3 shows the medical speciality the analyses were performed for. The majority of analyses were performed for innovations concerning Orthopedic Surgery (36, 10%), followed by Oncology (34, 9%) and Cardiology (30, 8%). Most analyses were on therapeutic innovations (269, 74%) compared to 93 (26%) analyses on diagnostic innovations and 2 innovations for prevention. Looking at the origin of the analyses, most were from the United States of America (111, 30%), followed by the United Kingdom (32, 9%) and the Netherlands (31, 9%). Most analyses were performed in high-income countries compared to low- and middle-income countries (305, 84% and 59, 16% respectively).

**Table 3:** Medical specialties of included analyses

	All Papers (*n* = 364) N	(%)
Orthopedic Surgery	36	(10)
Oncology	34	(9)
Cardiology	30	(8)
Neurology	27	(7)
Gastro Enterology	25	(7)
Infectious Diseases	20	(5)
Gynecology	16	(4)
Cardiothoracic Surgery	15	(4)
Vascular Surgery	15	(4)
Urology	13	(4)
Psychiatry	12	(3)
Radiotherapy	12	(3)
General Medicine	12	(3)
Pediatric Medicine	8	(2)
Genetics	7	(2)
Surgery	7	(2)
Ophthalmology	7	(2)
Endocrinology	6	(2)
Reproductive Health	6	(2)
Nephrology	5	(1)
Ear-Nose-Throat	5	(1)
Pulmonary Medicine	5	(1)
Radiology	5	(1)
Geriatrics	4	(1)
Hospital Medicine	4	(1)
Internal Medicine	4	(1)
Obstetrics	4	(1)
Plastic Surgery	4	(1)
Emergency Medicine	3	(1)
Neurosurgery	3	(1)
Intensive Care Medicine	2	(1)
Transplantation Surgery	2	(1)
Traumatology	2	(1)
Anesthesiology	1	(0.3)
Dental Care	1	(0.3)
Maxillofacial Surgery	1	(0.3)
Neonatology	1	(0.3)

### Description of methodologies

Table 4 shows the results of the methodological analysis. Out of 364 included papers, 268 (74%) contained cost-utility analyses and 91 (25%) cost-effectiveness analyses. A model was used in 236 (64%) analyses of which 107 were Markov models and 94 were Decision Trees and 22 microsimulation models. Thirteen analyses used a model but did not specify the type of model. A probabilistic sensitivity analysis (PSA) or bootstrapping in the case of trial-based analyses, were performed in 266 (73%) analyses, it was used less often in early analyses than late analyses, 9 (64%) and 257 (73%) respectively. Deterministic sensitivity analyses were used in 306 (84%) analyses, more often in early (12, 86%) than late analyses (294, 84%). Value of information analyses were performed in five (1%) analyses, three in early and two in late analyses. The perspective of the analyses was not reported in 16 (late) and 14 (early) per cent of papers. Of the studies that reported the perspective, early analyses employed a societal perspective more often, 36% versus 21%.

Of the 364 included papers the majority 350 (96%) were classed as late analyses, 243 (67%) were evaluations of innovations that were already implemented. Of late analyses, 115 (32%) were trial based, these analyses did not use a model and almost 3/4 of the trial-based analyses used bootstrapping (*n* = 86). Of the remaining 14 papers classed as early, 10 described analyses performed for innovations in a pre-clinical phase, the other four were performed in connection to a clinical trial, before a formal evaluation through the clinical trial. A larger proportion of early analyses used a model (11, 79%) compared to late analyses (225, 64%). Furthermore, early analyses used existing literature as input value sources more often than late analyses.

**Table 4:** Methodological analysis of included papers

	Early (*n* = 14)		Late (*n* = 350)		All (*n* = 364)	
	N	(%)	N	(%)	N	(%)
**Analysis**						
Cost Utility	11	(79)	257	(73)	268	(74)
Cost Effectiveness	3	(21)	88	(25)	91	(25)
Not Reported	0	(0)	4	(1)	4	(1)
Cost Minimization	0	(0)	1	(0)	1	(0)
**Model**						
No Model Used	3	(21)	125	(36)	128	(35)
Trial Based Economic Evaluations	2	(14)	115	(32)	117	(32)
Markov Model	7	(50)	100	(29)	107	(29)
Decision Tree	0	(0)	94	(27)	94	(26)
Microsimulation	3	(21)	19	(5)	22	(6)
Undetermined Model Used	1	(8)	12	(3)	13	(4)
**Perspective**						
Payer	7	(50)	201	(57)	208	(57)
Societal	5	(36)	75	(21)	80	(22)
Institutional	0	(0)	18	(5)	18	(5)
Not Reported	2	(14)	56	(16)	58	(16)
**Time horizon**						
Limited	10	(71)	194	(55)	204	(56)
Mean Time Horizon *(Months)*	*80*		*398*		*400*	
Lifelong	3	(21)	65	(19)	68	(19)
Not Reported	1	(8)	91	(26)	92	(25)
**Sensitivity analyses**						
Probabilistic/Bootstrap	9	(64)	257	(73)	266	(73)
Deterministic	12	(86)	294	(84)	306	(84)
**Value of Information Analyses**	3	(21)	2	(0)	5	(1)
**Source cost data**						
Hospital Database	1	(8)	72	(21)	73	(20)
Literature	5	(36)	101	(29)	106	(29)
Public Database	6	(42)	89	(25)	95	(26)
Clinical Research	2	(14)	79	(23)	81	(22)
Microcosting	0	(0)	3	(1)	3	(1)
Not Reported	0	(0)	6	(2)	6	(2)
**Source effectiveness data**						
Literature	10	(71)	189	(54)	199	(55)
Clinical Research	3	(21)	120	(35)	123	(34)
Hospital Database	0	(0)	21	(6)	21	(6)
Clinical Database	1	(8)	19	(5)	20	(5)
Not Reported	0	(0)	1	(0)	1	(0)
**Publication characteristics**						
CHEERS Declared by Authors	3	(21)	80	(23)	83	(23)
HEOR Journal	4	(29)	38	(11)	42	(12)
Clinical Journal	8	(57)	295	(84)	303	(83)
Other Journal	2	(14)	17	(5)	19	(5)

HEOR: Health Economics and Outcomes Research, CHEERS: Consolidated Health Economic Evaluation Reporting Standards

Subsequently, we investigated whether there was a difference in the methodology used and described in papers published in Health Economics and Outcomes Research (HEOR), clinical or other journals and whether a difference was found in papers declaring using the Consolidated Health Economic Evaluation Reporting Standards (CHEERS). Table 5 shows the results.

**Table 5:** Methodologies declared or used in different types of papers

	CHEERS Declared	PSA Used^A,B^	DSA Used^B^	VOI Used^B^	Perspective not reported^C^	Time horizon not reported^C^	Total
	N (%)	N (%)	N (%)	N (%)	N (%)	N (%)	N
**Journal type**							
Clinical	71 (23)	217 (72)	253 (83)	2 (0)	50 (17)	80 (26)	303
HEOR	7 (17)	36 (86)	40 (95)	3 (7)	3 (7)	8 (19)	42
Other	5 (26)	13 (68)	13 (68)	0 (0)	5 (26)	4 (21)	19
**CHEERS**							
Yes		74 (89)	78 (94)	2 (2)	3 (4)	9 (11)	83
No		192 (68)	228 (81)	3 (1)	55 (20)	82 (29)	281

HEOR: Health Economics and Outcomes Research, PSA: Probabilistic Sensitivity Analysis, DSA: Deterministic Sensitivity Analysis, VOI: Value of Information

^A^Including bootstrapping for trial-based analyses

^B^Higher percentages considered favorable

^C^Lower percentages considered favorable

## Discussion

The primary aim of this study was to provide insight into the types and extent of use of standard methodologies in the economic evaluation of medical technologies. From this study, we can conclude that the use of standard methodologies is still lacking in a substantial number of studies. Especially the inclusion of sensitivity analyses, Value of Information analyses and clear and consistent reporting were lacking. This underlines the importance of adherence to the guidelines, for execution as well as reporting as this improves the quality of analyses. Higher quality evaluations hold more value to the healthcare system as they could improve innovation quality as well as improve development quality. Compared to the number of late analyses included, the number of early economic evaluations was lower than expected.

More than half (65%) of analyses used decision analytic models, however, not all of them used (probabilistic) sensitivity analyses to account for model and parameter uncertainty. A PSA was performed in 179 (80%) of 225 model-based late analyses, and in 8 (73%) of 11 model-based early analyses. A deterministic sensitivity analysis was performed in 216 (96%) late and in 11 (100%) early model-based analyses. A lack of sensitivity analyses makes the critical appraisal of the model itself, the model outcomes and the data used more difficult and may lead to a reduction in the value of the analysis in the decision-making process, especially in early analyses. Furthermore, value of information (VOI) analyses, in which the value of additional research is quantified, were only used in five analyses, even though, especially in early evaluations, the VOI could be used to inform the next steps in the research and development process [11, 12]. The absence of VOI analyses could be explained by the perceived difficulty of the technique [11, 13]. More should be done to increase understandability, accessibility and usability of VOI to further expand the use of this type of analysis.

A total of 117 trial-based analyses were found in this mapping review, bootstrapping was used in three quarters (86, 74%) of these. A deterministic sensitivity analysis was performed in 84 (72%) of the trial-based evaluations. As trial-based analyses are performed alongside clinical trials, the time horizon of these analyses was often limited, 75 (64%). Only nine trial-based analyses were performed with a lifelong time horizon. Half of the model-based analyses, 129 of 247 (52%), were performed with a limited time horizon, 59 (24%) with a lifelong time horizon and for 59 (24%) the time horizon was not reported. The question is what a correct time horizon for analysis is, for example, the Dutch guideline recommends a lifelong time horizon for pharmaceuticals and leaves freedom to deviate for medical technologies whilst other guidelines and guidance papers suggest a time horizon sufficient enough to capture relevant costs and effects [14, 15].

As using standard methodologies is important for both early and late analyses, a lack of (probabilistic) sensitivity analyses, not using lifelong time horizons or not using the appropriate perspective could lead to decision makers not using analysis outcomes. This may eventually result in research waste or a delay in successful implementation of valuable innovations. A delay in implementation may result in a delay in healthcare improvement preventing opportunities to improve care for patients and reduce healthcare costs. Tailoring analyses to the needs of decision-makers is important. However, currently, it remains unclear if decision-makers have a different information need for different types of decisions and how this influences the choices for standard methodologies. Analyses in different phases of innovation development may be used by different types of stakeholders, each of which may use different decision criteria and thus may have different information needs. The information needs of different decision-makers should be taken into account when recommending or choosing methodologies for economic evaluations.

For many of the included papers it remains unclear how standard methodologies were implemented in the design due to insufficient reporting. For instance, perspective was not reported in 58 (16%) included studies and the time horizon was not reported in a quarter of included studies (*n* = 92 (25%)). Other included studies reported costs and effects in such a limited fashion, that reproduction of the analysis would not be feasible. The existence of the widely accepted CHEERS checklist did not lead to widespread use of these guidelines by authors (83, 23%) and it seems none of the guidelines or checklists were required by editors of journals [4]. Papers included in this review that declared using the CHEERS checklist used or reported standard methodologies better than papers that did not. Papers published in HEOR journals used or reported standard methodologies than papers published in other types of journals. However, papers published in clinical journals did declare using the CHEERS checklist more often.

Lack of use or reporting of standard methodologies could result in a reduction of the validity and trustworthiness of the analyses. This may ultimately lead to ineffective or non-use of results. We encourage all efforts to improve the knowledge of health economic methodologies, especially for those not deeply familiar with this area of research. Furthermore, the use of reporting checklists should be required by journal editors.

Our search resulted in a relatively low number of early analyses even though in 2022 performing early economic evaluations was a well-established method [16]. A reason for the absence of more early analyses could be that for medical technologies the innovations are still under development. The early economic evaluations should serve to inform the development process rather than serve as a formal evaluation of the technology [6, 17]. This could lead to a counter-incentive to publish these early economic analyses. The potential publication bias could not only hinder methodological research into early analyses but could also lead to research waste by preventing others from learning from failing innovations and thus failing fast and cheap [6]. Not only for the medical industry but also government subsidized device development, this could be an essential element to spending societal money efficiently.

Our second aim was to determine differences in standard methods used between early and late economic evaluations. We found that relatively more early analyses used decision analytic models (79%), compared to late analyses (64%). Apart from trial-based economic evaluations, a reason could be the limited availability of data on the exact costs and effects of the innovation in the early stages of development. The use of models allows for the linkage of data with different evidence levels whilst taking the uncertainty surrounding the linkage into account, allowing for health economic analyses to be made at these earlier stages [18]. The question remains whether the standard methodologies used in late analysis should be used as a blueprint for methodologies used in early evaluations.

A large part of late analyses used literature or clinical research as a basis for effect and/or cost estimation. Whether trial-based or literature-based analyses are more or less reliable and hold more or less value for decision-making compared to analyses using real-world data is to be determined. Given the nature of clinical trials, these protocols may not reflect routine care after implementation [19]. The reliability of analyses could be influenced by the correct use of standard methodologies. Furthermore, analyses using real-world data for effects and costs are also valuable for future early analyses since cost (-effectiveness) analyses in earlier stages often rely on data collected during trials.

A striking observation in some of the papers excluded from this analysis (*n* = 48) was that authors used the term “cost-effectiveness analysis” or “economic evaluation” for any analysis in which clinical effects were offset against hospital charges. We would like to encourage authors and editors of journals to use these terms only when appropriate, validated and established health economic methods are used.

### Strengths and limitations of this review

A strength of this review is the number of papers included in the analysis, this in combination with the search strategy ensures the inclusion of a wide range of methodologies used in health economic research included in the analysis. As this is a mapping review no critical appraisal of included papers was performed [10]. This can be seen as a limitation, but a critical appraisal was not performed as our focus was to map the different methodologies used and not to evaluate the quality of the research performed.

## Conclusions

This review shows the wide range of methodologies used in economic evaluations as well as the extent to which these methodologies are used. Many of the included papers did not use or did not sufficiently report the use of standard methods leading to a lack of trustworthiness in outcomes and maybe even validity of outcomes. This may lead to research waste, a delay in the successful implementation of valuable innovations and subsequently a delay in the improvement of care for the patient. Reporting should be improved by requiring the use of checklists by journals and efforts should be made to encourage parties to publish early economic evaluations.

### Electronic supplementary material

Below is the link to the electronic supplementary material.

Search String and References of Included Papers

## Data Availability

Data Extraction files and Rayyan files available at the corresponding author.
