# Supplementary material for: Economic evaluations in medical technological innovations a mapping review of methodologies
Source: Cost Eff Resour Alloc. 2024 Mar 19;22:23. doi: 10.1186/s12962-024-00529-0 (PMC10953233; doi:10.1186/s12962-024-00529-0)
Supplement: Supplementary file 1 — Search String and References of Included Papers [file 12962_2024_529_MOESM1_ESM.docx]

Economic Evaluations in Medical Technological Innovations

A Mapping Review of Methodologies

C. van Lieshout^1,2^, G.W.J. Frederix^1,2^, L. Schoonhoven^1^

**Supplemental Materials**

- 1. PUBMED-search string 3-01-2024

| No | Terms | Hits |
| --- | --- | --- |
| 1 | "cost benefit"[Title/Abstract] OR "cost-effectiveness"[Title/Abstract] OR "cost-utility"[Title/Abstract] OR "economic evaluation"[Title/Abstract] OR "cost-utility"[Title/Abstract] OR "technology assessment"[Title/Abstract] OR "cost-effectiveness"[Title/Abstract] | 104,421 |
| 2 | #1 AND ONLY 2022 | 7,735 |
| 3 | #2 AND NO PREPRINTS | 7,734 |
| 4 | #3 AND ONLY IN HUMANS | 5,173 |
| 5 | #4 AND ONLY DUTCH OR ENGLISH | **5,079** |

- 1. References of included papers

S-1. A Alcaraz, A Pichon-Riviere, C Rojas-Roque, JM González, D Prina, G Solioz, et al. Cost-effectiveness of a centrifugal-flow pump for patients with advanced heart failure in Argentina. PloS one. 2022;17(8):e0271519.

S-2. A Atik, ET Fahy, LA Rhodes, BC Samuels, ST Mennemeyer, CA Girkin. Comparative Cost-effectiveness of Trabeculectomy versus MicroShunt in the US Medicare System. Ophthalmology. 2022;129(10):1142-51.

S-3. A Allou, L Baschet, C Sabourin, G Montalscot, L Lorgis, X Iriart. Cost-effectiveness analysis of patent foramen ovale closure with Amplatzer plus medical therapy compared to medical therapy in patients with a history of stroke in France. Journal of cardiology. 2022;80(1):72-9.

S-4. A Baka, T van der Zweerde, J Lancee, JE Bosmans, A van Straten. Cost-effectiveness of Guided Internet-Delivered Cognitive Behavioral Therapy in Comparison with Care-as-Usual for Patients with Insomnia in General Practice. Behavioral sleep medicine. 2022;20(2):188-203.

S-5. A Cernat, M Jamieson, R Kavelaars, S Khalili, V Bhambhwani, K Mireskandari, ME Moretti. Immediate versus delayed sequential bilateral cataract surgery in children: a cost-effectiveness analysis. The British journal of ophthalmology. 2022;106(2):211-7.

S-6. A Darvishi, P Sadeghipour, A Darrudi, R Daroudi. Cost-utility analysis of Cryoballoon ablation versus Radiofrequency ablation in the treatment of paroxysmal atrial fibrillation in Iran. PloS one. 2022;17(7):e0270642.

S-7. A Engelgeer, A van Westrhenen, RD Thijs, SMAA Evers. An economic evaluation of the NightWatch for children with refractory epilepsy: Insight into the cost-effectiveness and cost-utility. Seizure. 2022;101:156-61.

S-8. A Gandjour. Benefits, risks, and cost-effectiveness of COVID-19 self-tests from a consumer's perspective. BMC health services research. 2022;22(1):47.

S-9. A Jameel, A Meiwald, P Bain, N Patel, D Nandi, B Jones, et al. The cost-effectiveness of unilateral magnetic resonance-guided focused ultrasound in comparison with unilateral deep brain stimulation for the treatment of medically refractory essential tremor in England. The British journal of radiology. 2022;95(1140):20220137.

S-10. A Joeris, M Kabiri, T Galvain, M Vanderkarr, CE Holy, JQ Plaza, et al. Cost-Effectiveness of Cement Augmentation Versus No Augmentation for the Fixation of Unstable Trochanteric Fractures. The Journal of bone and joint surgery American volume. 2022;104(22):2026-34.

S-11. A Kuhlmann, K Hagberg, I Kamrad, N Ramstrand, S Seidinger, H Berg. The Kenevo microprocessor-controlled prosthetic knee compared with non-microprocessor-controlled knees in individuals older than 65 years in Sweden: A cost-effectiveness and budget-impact analysis. Prosthetics and orthotics international. 2022;46(5):414-24.

S-12. A Le Bras, E Puymirat, H Rabetrano, G Cayla, T Simon, G Steg, et al. Economic evaluation of fractional flow reserve-guided versus angiography-guided multivessel revascularisation in ST-segment elevation myocardial infarction patients in the FLOWER-MI randomised trial. EuroIntervention : journal of EuroPCR in collaboration with the Working Group on Interventional Cardiology of the European Society of Cardiology. 2022;18(3):235-41.

S-13. A Marcellusi, FS Mennini, M Ruf, C Galli, A Aghemo, MR Brunetto, et al. Optimizing diagnostic algorithms to advance Hepatitis C elimination in Italy: A cost effectiveness evaluation. Liver international : official journal of the International Association for the Study of the Liver. 2022;42(1):26-37.

S-14. A Makki, JB Thomsen, GL Gunnarsson, PLR Hölmich, PJA Sørensen, MB Rindom. A cost-effectiveness analysis of delayed breast reconstruction with pedicled flaps from the back. Journal of plastic, reconstructive & aesthetic surgery : JPRAS. 2022;75(7):2211-8.

S-15. A Mujcic, M Blankers, B Boon, AH Berman, H Riper, M van Laar, R Engels. Effectiveness, Cost-effectiveness, and Cost-Utility of a Digital Alcohol Moderation Intervention for Cancer Survivors: Health Economic Evaluation and Outcomes of a Pragmatic Randomized Controlled Trial. Journal of medical Internet research. 2022;24(2):e30095.

S-16. A Pease, E Callander, E Zomer, MB Abraham, EA Davis, TW Jones, et al. The Cost of Control: Cost-effectiveness Analysis of Hybrid Closed-Loop Therapy in Youth. Diabetes care. 2022;45(9):1971-80.

S-17. A Peacock, F Dehle, OA Mesa Zapata, HM Prince, F Gennari, C Taylor. Cost-Effectiveness of Extracorporeal Photopheresis for the Treatment of Patients With Erythrodermic (Stage T(4), M(0)) Cutaneous T-Cell Lymphoma in the Australian Setting. Value in health : the journal of the International Society for Pharmacoeconomics and Outcomes Research. 2022;25(6):965-74.

S-18. A Ribera, E Giménez, G Oristrell, D Osorio, JR Marsal, L García-Pérez, et al. Cost-effectiveness of implantable cardioverter-defibrillators for primary prevention of sudden cardiac death. Revista espanola de cardiologia (English ed). 2022;75(1):12-21.

S-19. A Vaiarelli, D Cimadomo, G Gennarelli, M Guido, C Alviggi, A Conforti, et al. Second stimulation in the same ovarian cycle: an option to fully-personalize the treatment in poor prognosis patients undergoing PGT-A. Journal of assisted reproduction and genetics. 2022;39(3):663-73.

S-20. A Werbrouck, M Schmidt, L Annemans, J Duyck, B Janssens, S Simoens, et al. Oral healthcare delivery in institutionalised older people: A health-economic evaluation. Gerodontology. 2022;39(2):107-20.

S-21. A Zemplényi, J Józwiak-Hagymásy, S Kovács, D Erdősi, I Boncz, T Tényi, et al. Repetitive transcranial magnetic stimulation may be a cost-effective alternative to antidepressant therapy after two treatment failures in patients with major depressive disorder. BMC psychiatry. 2022;22(1):437.

S-22. AA Ali, R Tawk, H Xiao, A Semykina, AJ Montero, RK Moussa, et al. Comparative cost-effectiveness of radiotherapy among older women with hormone receptor positive early-stage breast cancer. Expert review of pharmacoeconomics & outcomes research. 2022;22(5):735-41.

S-23. AB Ross, A Jacobs, KL Williams, RK Bour, S Gyftopoulos. Ultrasound-Guided Injection Treatments Versus Surgical Neurectomy for Morton Neuroma: A Cost-Effectiveness Analysis. United States2022 2022-2. 234-40 p.

S-24. AC Lo, LP James, A Prica, A Raymakers, S Peacock, M Qu, et al. PET-Based Staging Is Cost-Effective in Early-Stage Follicular Lymphoma. Journal of nuclear medicine : official publication, Society of Nuclear Medicine. 2022;63(4):543-8.

S-25. AJ Pease, S Zoungas, E Callander, TW Jones, SR Johnson, DJ Holmes-Walker, et al. Nationally Subsidized Continuous Glucose Monitoring: A Cost-effectiveness Analysis. Diabetes care. 2022;45(11):2611-9.

S-26. AMM Loohuis, H Van Der Worp, NJ Wessels, JH Dekker, MCP Slieker-Ten Hove, MY Berger, et al. Cost-effectiveness of an app-based treatment for urinary incontinence in comparison with care-as-usual in Dutch general practice: a pragmatic randomised controlled trial over 12 months. BJOG : an international journal of obstetrics and gynaecology. 2022;129(9):1538-45.

S-27. AN Webb, ELW Lester, AMJ Shapiro, DT Eurich, DL Bigam. Cost-utility analysis of normothermic machine perfusion compared to static cold storage in liver transplantation in the Canadian setting. American journal of transplantation : official journal of the American Society of Transplantation and the American Society of Transplant Surgeons. 2022;22(2):541-51.

S-28. AS Schuit, K Holtmaat, VMH Coupé, SEJ Eerenstein, JM Zijlstra, C Eeltink, et al. Cost-Utility of the eHealth Application 'Oncokompas', Supporting Incurably Ill Cancer Patients to Self-Manage Their Cancer-Related Symptoms: Results of a Randomized Controlled Trial. Current oncology (Toronto, Ont). 2022;29(9):6186-202.

S-29. AV Hernández, J Otten, H Christ, C Ulrici, E Piriyev, S Ludwig, C Rudroff. Ghost Ileostomy: Safe and Cost-effective Alternative to Ileostomy After Rectal Resection for Deep Infiltrating Endometriosis. Greece2022 2022-5. 1290-6 p.

S-30. AW Blom, E Lenguerrand, S Strange, SM Noble, AD Beswick, A Burston, et al. Clinical and cost effectiveness of single stage compared with two stage revision for hip prosthetic joint infection (INFORM): pragmatic, parallel group, open label, randomised controlled trial. BMJ (Clinical research ed). 2022;379:e071281.

S-31. B Dawkins, N Aruparayil, T Ensor, J Gnanaraj, J Brown, D Jayne, B Shinkins. Cost-effectiveness of gasless laparoscopy as a means to increase provision of minimally invasive surgery for abdominal conditions in rural North-East India. PloS one. 2022;17(8):e0271559.

S-32. B Niu, N Mukhtarova, O Alagoz, K Hoppe. Cost-effectiveness of telehealth with remote patient monitoring for postpartum hypertension. England2022 2022-12. 7555-61 p.

S-33. B Te Ao, M Harwood, V Fu, M Weatherall, K McPherson, WJ Taylor, et al. Economic analysis of the 'Take Charge' intervention for people following stroke: Results from a randomised trial. Clinical rehabilitation. 2022;36(2):240-50.

S-34. B Wayda, XS Cheng, JD Goldhaber-Fiebert, KK Khush. Optimal patient selection for simultaneous heart-kidney transplant: A modified cost-effectiveness analysis. American journal of transplantation : official journal of the American Society of Transplantation and the American Society of Transplant Surgeons. 2022;22(4):1158-68.

S-35. BE Nichols, A de Nooy, M Benade, K Balakasi, M Mphande, G Rao, et al. Facility-based HIV self-testing strategies may substantially and cost-effectively increase the number of men and youth tested for HIV in Malawi: results from an individual-based mathematical model. Journal of the International AIDS Society. 2022;25(10):e26020.

S-36. BF Dear, E Karin, R Fogliati, J Dudeney, O Nielssen, M Gandy, et al. The Pain Course: a randomised controlled trial and economic evaluation of an internet-delivered pain management program. Pain. 2022;163(7):1388-401.

S-37. BK Yoo, NH Yang, K Hoffman, T Sasaki, SC Haynes, J Mouzoon, JP Marcin. Economic Evaluation of Telemedicine Consultations to Reduce Unnecessary Neonatal Care Transfers. The Journal of pediatrics. 2022;244:58-63.e1.

S-38. BN Lauren, F Lim, A Krikhely, EM Taveras, JA Woo Baidal, BK Bellows, C Hur. Estimated Cost-effectiveness of Medical Therapy, Sleeve Gastrectomy, and Gastric Bypass in Patients With Severe Obesity and Type 2 Diabetes. JAMA network open. 2022;5(2):e2148317.

S-39. BT Heiden, JD Mitchell, E Rome, V Puri, BF Meyers, SH Chang, BD Kozower. Cost-Effectiveness Analysis of Robotic-assisted Lobectomy for Non-Small Cell Lung Cancer. The Annals of thoracic surgery. 2022;114(1):265-72.

S-40. BW Risør, N Tayyari Dehbarez, J Fredsøe, KD Sørensen, BG Pedersen. Cost-Effectiveness Analysis of Stockholm 3 Testing Compared to PSA as the Primary Blood Test in the Prostate Cancer Diagnostic Pathway: A Decision Tree Approach. Applied health economics and health policy. 2022;20(6):867-80.

S-41. C Bommer, J Lupatsch, N Bürki, M Schwenkglenks. Cost-utility analysis of risk-reducing strategies to prevent breast and ovarian cancer in BRCA-mutation carriers in Switzerland. The European journal of health economics : HEPAC : health economics in prevention and care. 2022;23(5):807-21.

S-42. C Gayot, C Laubarie-Mouret, K Zarca, M Mimouni, N Cardinaud, S Luce, et al. Effectiveness and cost-effectiveness of a telemedicine programme for preventing unplanned hospitalisations of older adults living in nursing homes: the GERONTACCESS cluster randomized clinical trial. BMC geriatrics. 2022;22(1):991.

S-43. C Grobet, L Audigé, K Eichler, F Meier, M Marks, DB Herren. Cost-Utility Analysis of Thumb Carpometacarpal Resection Arthroplasty: A Health Economic Study Using Real-World Data. The Journal of hand surgery. 2022;47(5):445-53.

S-44. C Hanlon, G Medhin, ME Dewey, M Prince, E Assefa, T Shibre, et al. Efficacy and cost-effectiveness of task-shared care for people with severe mental disorders in Ethiopia (TaSCS): a single-blind, randomised, controlled, phase 3 non-inferiority trial. England2022 2022-1. 59-71 p.

S-45. C Handford, L McMenemy, J Kendrew, A Mistlin, MA Akhtar, M Parry, P Hindle. Improving outcomes for amputees: The health-related quality of life and cost utility analysis of osseointegration prosthetics in transfemoral amputees. Injury. 2022;53(12):4114-22.

S-46. C Henderson, M Knapp, J Fossey, E Frangou, C Ballard. Cost-Effectiveness of an Online Intervention for Caregivers of People Living With Dementia. United States; 2022 2022-9. Report No.: 1538-9375 (Electronic) Contract No.: 9.

S-47. CA Veet, S Capone, D Panczykowski, N Parekh, KJ Smith, DH Kim, et al. Imaging versus Intervention in Managing Small Unruptured Intracranial Aneurysms: A Cost-Effectiveness Analysis. Cerebrovascular diseases (Basel, Switzerland). 2022;51(3):338-48.

S-48. CCA Lau, F Irani, L Shi, A Patel, KD Zhuang, S Chandramohan, et al. Cost-Effectiveness of Drug-Coated Balloon Angioplasty Compared With Conventional Balloon Angioplasty for Arteriovenous Access Flow Dysfunction. Value in health regional issues. 2022;31:155-62.

S-49. CE Rodríguez-Martínez, MP Sossa-Briceño, G Nino. Emergency department-initiated home oxygen for viral bronchiolitis: A cost-effectiveness analysis. Pediatric pulmonology. 2022;57(9):2154-60.

S-50. CE Rodriguez-Martinez, MP Sossa-Briceño, J Antonio Buendia. Comparison of two oxygen saturation targets to decide on hospital discharge of infants with viral bronchiolitis living at high altitudes: a cost-effectiveness analysis. Current medical research and opinion. 2022;38(12):2047-53.

S-51. CG Fawsitt, D Lucey, P Harrington, K Jordan, L Marshall, KK O'Brien, C Teljeur. A cost-effectiveness and budget impact analysis of C-reactive protein point-of-care testing to guide antibiotic prescribing for acute respiratory tract infections in primary care settings in Ireland: a decision-analytic model. Family practice. 2022;39(3):389-97.

S-52. CH Packer, AR Hersh, JA Sargent, AB Caughey. Therapeutic hypothermia in severe hypoxic-ischemic encephalopathy: a cost-effectiveness analysis. The journal of maternal-fetal & neonatal medicine : the official journal of the European Association of Perinatal Medicine, the Federation of Asia and Oceania Perinatal Societies, the International Society of Perinatal Obstetricians. 2022;35(5):890-7.

S-53. CJ Magnani, T Hernandez-Boussard, LC Baker, JD Goldhaber-Fiebert, JD Brooks. Cost-Effectiveness Analysis and Microsimulation of Serial Multiparametric Magnetic Resonance Imaging in Active Surveillance of Localized Prostate Cancer. The Journal of urology. 2022;208(1):80-9.

S-54. CL Cui, WY Luo, BC Cosman, S Eisenstein, D Simpson, S Ramamoorthy, et al. Cost Effectiveness of Watch and Wait Versus Resection in Rectal Cancer Patients with Complete Clinical Response to Neoadjuvant Chemoradiation. Annals of surgical oncology. 2022;29(3):1894-907.

S-55. CM Avram, AB Caughey, ME Norton, TN Sparks. Cost-Effectiveness of Exome Sequencing versus Targeted Gene Panels for Prenatal Diagnosis of Fetal Effusions and Non-Immune Hydrops Fetalis. American journal of obstetrics & gynecology MFM. 2022;4(6):100724.

S-56. CP Smith, B Adefres, EM Chang, TQ Huang, N Parikh, A Raldow. Cost-Effectiveness of PET Directed Versus Combined Modality Therapy for Early-Stage Favorable Hodgkin's Lymphoma. Clinical lymphoma, myeloma & leukemia. 2022;22(11):e992-e9.

S-57. CR Mayhew, A Gallagher, A Bensimhon, HL Dauerman, MH Tsai, JA Martin. Cost-effectiveness analysis of a resource-intensive approach versus minimally invasive strategy for high-risk transcatheter aortic valve replacement patients. Journal of comparative effectiveness research. 2022;11(4):217-27.

S-58. CS Lam, PK Dhedli, S Russell, FE Stedman, NJ Hall. Cost-Effectiveness of Laparoscopic and Open Pediatric Inguinal Hernia Repair. Journal of laparoendoscopic & advanced surgical techniques Part A. 2022;32(7):805-10.

S-59. CSS Latenstein, SZ Wennmacker, AH van Dijk, JPH Drenth, GP Westert, CJHM van Laarhoven, et al. Cost-effectiveness of Restrictive Strategy Versus Usual Care for Cholecystectomy in Patients With Gallstones and Abdominal Pain (SECURE-trial). Annals of surgery. 2022;276(2):e93-e101.

S-60. CTJ Michels, CJ Wijburg, G Hannink, JA Witjes, MM Rovers, JPC Grutters. Robot-assisted Versus Open Radical Cystectomy in Bladder Cancer: An Economic Evaluation Alongside a Multicentre Comparative Effectiveness Study. European urology focus. 2022;8(3):739-47.

S-61. D Al-Badriyeh, AA Hssain, D Abushanab. Cost-Effectiveness Analysis of Out-Of-Hospital versus In-Hospital Extracorporeal Cardiopulmonary Resuscitation for Out-Hospital Refractory Cardiac Arrest. Current problems in cardiology. 2022;47(12):101387.

S-62. D Charleux-Muller, B Romain, C Boisson, M Velten, C Brigand, C Lejeune. Cost-effectiveness analysis of resorbable biosynthetic mesh in contaminated ventral hernia repair. Journal of visceral surgery. 2022;159(4):279-85.

S-63. D Franzen, C Bodmer, S Ehrenbaum, C Steinack, I Opitz, K Docter, O Schöffski. Cost-effectiveness analysis of surgical lung volume reduction compared with endobronchial valve treatment in patients with severe emphysema. Swiss medical weekly. 2022;152:40008.

S-64. D Fernández-Sanchis, MP López-Royo, C Jiménez-Sánchez, P Herrero, M Gómez-Barrera, S Calvo. A comparative study of treatment interventions for patellar tendinopathy: a secondary cost-effectiveness analysis. England2022 2022-12. 516-23 p.

S-65. D Fernández Sanchis, JN Cuenca Zaldívar, S Calvo, P Herrero, M Gómez Barrera. Cost-effectiveness of upper extremity dry needling in the rehabilitation of patients with stroke. Acupuncture in medicine : journal of the British Medical Acupuncture Society. 2022;40(2):160-8.

S-66. D Lewkowicz, AM Wohlbrandt, E Bottinger. Digital Therapeutic Care Apps With Decision-Support Interventions for People With Low Back Pain in Germany: Cost-Effectiveness Analysis. JMIR mHealth and uHealth. 2022;10(2):e35042.

S-67. D Mehta, AH Loutfy, VM Kushnir, AL Faulx, ZL Smith. Cold versus hot endoscopic mucosal resection for large sessile colon polyps: a cost-effectiveness analysis. Endoscopy. 2022;54(4):367-75.

S-68. D Nagendra, SM Gutman, NC Koelper, SE Loza-Avalos, S Sonalkar, CA Schreiber, HS Harvie. Medical management of early pregnancy loss is cost-effective compared with office uterine aspiration. American journal of obstetrics and gynecology. 2022;227(5):737.e1-.e11.

S-69. D Pollard, G Fuller, S Goodacre, EAJ van Rein, JF Waalwijk, M van Heijl. An economic evaluation of triage tools for patients with suspected severe injuries in England. BMC emergency medicine. 2022;22(1):4.

S-70. D Puhr-Westerheide, MF Froelich, O Solyanik, E Gresser, P Reidler, MP Fabritius, et al. Cost-effectiveness of short-protocol emergency brain MRI after negative non-contrast CT for minor stroke detection. European radiology. 2022;32(2):1117-26.

S-71. D Raimondo, I Giaquinto, M Maletta, R Vicenti, R Iodice, A Arena, et al. Cost-effectiveness analysis of ovarian tissue cryopreservation and transplantation for preservation of fertility in post-pubertal oncological women submitted to high-risk gonadotoxic chemotherapy. International journal of gynaecology and obstetrics: the official organ of the International Federation of Gynaecology and Obstetrics. 2022;159(1):116-21.

S-72. D Yeroushalmi, J Feng, L Nherera, P Trueman, R Schwarzkopf. Early Economic Analysis of Robotic-Assisted Unicondylar Knee Arthroplasty May Be Cost Effective in Patients with End-Stage Osteoarthritis. The journal of knee surgery. 2022;35(1):39-46.

S-73. D Zou, W Ye, LM Hess, NR Bhandari, A Ale-Ali, J Foster, et al. Diagnostic Value and Cost-Effectiveness of Next-Generation Sequencing-Based Testing for Treatment of Patients with Advanced/Metastatic Non-Squamous Non-Small-Cell Lung Cancer in the United States. The Journal of molecular diagnostics : JMD. 2022;24(8):901-14.

S-74. DD Bu, ZG Schwam, VF Kaul, K Wong, C Fan, GB Wanna, et al. Cost-effectiveness of Canal Wall-Up vs Canal Wall-Down Mastoidectomy: A Modeling Study. England; 2022 2022-9. Report No.: 1097-6817 (Electronic) Contract No.: 3.

S-75. DH Smith, M O'Keeffe-Rosetti, SL Fitzpatrick, M Mayhew, AJ Firemark, I Gruß, et al. Costs and Cost-Effectiveness of Implementing a Digital Diabetes Prevention Program in a Large, Integrated Health System. The Permanente journal. 2022;26(3):74-82.

S-76. DJ Cohen, K Wang, E Magnuson, R Smith, MC Petrie, MH Buch, et al. Cost-effectiveness of transcatheter edge-to-edge repair in secondary mitral regurgitation. Heart (British Cardiac Society). 2022;108(9):717-24.

S-77. DJ Cunningham, TS Pidgeon, EB Saltzman, RC Mather, DS Ruch. The Value Added of Advanced Imaging in the Diagnosis and Treatment of Triangular Fibrocartilage Complex Pathology. The Journal of hand surgery. 2022;47(1):19-30.e8.

S-78. DL Ejalu, A Irioko, R Kirabo, AD Mukose, E Ekirapa, J Kagaayi, J Namutundu. Cost-effectiveness of GeneXpert Omni compared with GeneXpert MTB/Rif for point-of-care diagnosis of tuberculosis in a low-resource, high-burden setting in Eastern Uganda: a cost-effectiveness analysis based on decision analytical modelling. BMJ open. 2022;12(8):e059823.

S-79. DS Chew, PA Cowper, H Al-Khalidi, KJ Anstrom, MR Daniels, L Davidson-Ray, et al. Cost-Effectiveness of Coronary Artery Bypass Surgery Versus Medicine in Ischemic Cardiomyopathy: The STICH Randomized Clinical Trial. Circulation. 2022;145(11):819-28.

S-80. DS Chew, Y Li, PA Cowper, KJ Anstrom, JP Piccini, JE Poole, et al. Cost-Effectiveness of Catheter Ablation Versus Antiarrhythmic Drug Therapy in Atrial Fibrillation: The CABANA Randomized Clinical Trial. Circulation. 2022;146(7):535-47.

S-81. DV Pachito, ALF de Azeredo-da-Silva, PRBP de Oliveira, ÂM Bagattini, J Basso, LG Gehres, et al. Telehealth Strategies to Support Referral Management to Secondary Care in Brazil: A Cost-Effectiveness Analysis. Value in health regional issues. 2022;31:74-80.

S-82. E Burian, B Palla, N Callahan, T Pyka, C Wolff, CE von Schacky, et al. Comparison of CT, MRI, and F-18 FDG PET/CT for initial N-staging of oral squamous cell carcinoma: a cost-effectiveness analysis. European journal of nuclear medicine and molecular imaging. 2022;49(11):3870-7.

S-83. E de Alava, MJ Pareja, D Carcedo, N Arrabal, JF García, R Bernabé-Caro. Cost-effectiveness analysis of molecular diagnosis by next-generation sequencing versus sequential single testing in metastatic non-small cell lung cancer patients from a south Spanish hospital perspective. Expert review of pharmacoeconomics & outcomes research. 2022;22(6):1033-42.

S-84. E González Diaz, C Rodríguez-Paz, A Fernandez-Prieto, M Martínez-Galdámez, R Martínez-Moreno, J Ortega Quintanilla, et al. Economic impact of the first pass effect in mechanical thrombectomy for acute ischaemic stroke treatment in Spain: a cost-effectiveness analysis from the national health system perspective. BMJ open. 2022;12(9):e054816.

S-85. E Lee, J Zhang. Which assisted reproductive technology (ART) treatment strategy is the most clinically and cost-effective for women of advanced maternal age: a Markov model. BMC health services research. 2022;22(1):1197.

S-86. E Nivelle, S Dewilde, A Peeters, G Vanhooren, V Thijs. Thrombectomy is a cost-saving procedure up to 24 h after onset. Acta neurologica Belgica. 2022;122(1):163-71.

S-87. E Otieku, AP Fenny, FA Asante, A Bediako-Bowan, U Enemark. Cost-effectiveness analysis of an active 30-day surgical site infection surveillance at a tertiary hospital in Ghana: evidence from HAI-Ghana study. BMJ open. 2022;12(1):e057468.

S-88. E Pinar, J García de Lara, J Hurtado, M Robles, G Leithold, B Martí-Sánchez, et al. Cost-effectiveness analysis of the SAPIEN 3 transcatheter aortic valve implant in patients with symptomatic severe aortic stenosis. Revista espanola de cardiologia (English ed). 2022;75(4):325-33.

S-89. EA Magnuson, K Chinnakondepalli, K Vilain, PW Serruys, JF Sabik, AP Kappetein, et al. Cost-Effectiveness of Percutaneous Coronary Intervention Versus Bypass Surgery for Patients With Left Main Disease: Results From the EXCEL Trial. Circulation Cardiovascular interventions. 2022;15(7):e011981.

S-90. EF Faria, RP Rosim, E de Matos Nogueira, M Tobias-Machado. Cost-Effectiveness Analysis of Robotic-Assisted Radical Prostatectomy for Localized Prostate Cancer From the Brazilian Public System Perspective. Value in health regional issues. 2022;29:60-5.

S-91. EF Nash, J Choyce, V Carrolan, E Justice, KL Shaw, A Sitch, et al. A prospective randomised controlled mixed-methods pilot study of home monitoring in adults with cystic fibrosis. Therapeutic advances in respiratory disease. 2022;16:17534666211070133.

S-92. EH Serné, S Roze, MI Buompensiere, WJ Valentine, S De Portu, HW de Valk. Cost-Effectiveness of Hybrid Closed Loop Insulin Pumps Versus Multiple Daily Injections Plus Intermittently Scanned Glucose Monitoring in People With Type 1 Diabetes in The Netherlands. Advances in therapy. 2022;39(4):1844-56.

S-93. EI Howe, N Andelic, SCR Fure, C Røe, HL Søberg, T Hellstrøm, et al. Cost-effectiveness analysis of combined cognitive and vocational rehabilitation in patients with mild-to-moderate TBI: results from a randomized controlled trial. BMC health services research. 2022;22(1):185.

S-94. EJ de Koster, D Vriens, MO van Aken, LT Dijkhorst-Oei, WJG Oyen, RP Peeters, et al. FDG-PET/CT in indeterminate thyroid nodules: cost-utility analysis alongside a randomised controlled trial. European journal of nuclear medicine and molecular imaging. 2022;49(10):3452-69.

S-95. EK Donovan, F Xie, AV Louie, W Chu, S Siva, A Kapoor, A Swaminath. Cost Effectiveness Analysis of Radiofrequency Ablation (RFA) Versus Stereotactic Body Radiotherapy (SBRT) for Early Stage Renal Cell Carcinoma (RCC). Clinical genitourinary cancer. 2022;20(5):e353-e61.

S-96. EL Yang, PT Levy, PJ Critser, D Dukhovny, PD Evers. The Clinical and Cost Utility of Cardiac Catheterizations in Infants with Bronchopulmonary Dysplasia. The Journal of pediatrics. 2022;246:56-63.e3.

S-97. EM Camacho, S Whyte, SJ Stock, CJ Weir, JE Norman, AEP Heazell. Awareness of fetal movements and care package to reduce fetal mortality (AFFIRM): a trial-based and model-based cost-effectiveness analysis from a stepped wedge, cluster-randomised trial. BMC pregnancy and childbirth. 2022;22(1):235.

S-98. ES Aby, BP Vaughn, EA Enns, R Rajasingham. Cost-effectiveness of Fecal Microbiota Transplantation for First Recurrent Clostridioides difficile Infection. Clinical infectious diseases : an official publication of the Infectious Diseases Society of America. 2022;75(9):1602-9.

S-99. F Alarid-Escudero, D Schrag, KM Kuntz. CDX2 Biomarker Testing and Adjuvant Therapy for Stage II Colon Cancer: An Exploratory Cost-Effectiveness Analysis. Value in health : the journal of the International Society for Pharmacoeconomics and Outcomes Research. 2022;25(3):409-18.

S-100. F Labori, J Persson, C Bonander, K Jood, M Svensson. Cost-effectiveness analysis of left atrial appendage occlusion in patients with atrial fibrillation and contraindication to oral anticoagulation. European heart journal. 2022;43(13):1348-56.

S-101. F Machleid, J Ho-Wrigley, A Chowdhury, A Paliah, HL Poon, E Pizzo. Cost-utility analysis of robotic-assisted radical cystectomy for bladder cancer compared to open radical cystectomy in the United Kingdom. PloS one. 2022;17(9):e0270368.

S-102. F Monteiro, P Antunes, M Pereira, MC Canavarro, A Fonseca. Cost-utility of a web-based intervention to promote maternal mental health among postpartum women presenting low risk for postpartum depression. International journal of technology assessment in health care. 2022;38(1):e62.

S-103. F Meethale Thiruvoth, SR Rajasulochana, MK S, S E, P Sivanantham, SS Kar. Hyperbaric oxygen therapy as an adjunct to the standard wound care for the treatment of diabetic foot ulcers in Indian patients: a cost utility analysis. Expert review of pharmacoeconomics & outcomes research. 2022;22(7):1087-94.

S-104. F Schwendicke, S Mertens, AG Cantu, A Chaurasia, H Meyer-Lueckel, J Krois. Cost-effectiveness of AI for caries detection: randomized trial. Journal of dentistry. 2022;119:104080.

S-105. FG Gassert, S Ziegelmayer, J Luitjens, FT Gassert, F Tollens, J Rink, et al. Additional MRI for initial M-staging in pancreatic cancer: a cost-effectiveness analysis. European radiology. 2022;32(4):2448-56.

S-106. FJ Gilbert, S Harris, KA Miles, JR Weir-McCall, NR Qureshi, RC Rintoul, et al. Comparative accuracy and cost-effectiveness of dynamic contrast-enhanced CT and positron emission tomography in the characterisation of solitary pulmonary nodules. Thorax. 2022;77(10):988-96.

S-107. FM Kuijper, UV Mahajan, S Ku, DAN Barbosa, SM Alessi, SC Stein, et al. Deep Brain Stimulation Compared With Contingency Management for the Treatment of Cocaine Use Disorders: A Threshold and Cost-Effectiveness Analysis. Neuromodulation : journal of the International Neuromodulation Society. 2022;25(2):253-62.

S-108. FS Ali, T DaVee, EV Bernstam, LS Kao, M Wandling, MR Hussain, et al. Cost-effectiveness analysis of optimal diagnostic strategy for patients with symptomatic cholelithiasis with intermediate probability for choledocholithiasis. Gastrointestinal endoscopy. 2022;95(2):327-38.

S-109. FS Mennini, F Meucci, G Pesarini, P Vandoni, M Lettino, A Sarmah, et al. Cost-effectiveness of transcatheter aortic valve implantation versus surgical aortic valve replacement in low surgical risk aortic stenosis patients. International journal of cardiology. 2022;357:26-32.

S-110. G Bediang, CN Nganou-Gnindjio, Y Kamga, JS Ndongo, FC Goethe Doualla, CO Bagayoko, S Nko'o. Evaluation of the Efficiency of Telemedicine in the Management of Cardiovascular Diseases in Primary Healthcare in Sub-Saharan Africa: A Medico-Economic Study in Cameroon. Studies in health technology and informatics. 2022;294:910-4.

S-111. G Fekadu, J Yao, JHS You. Cost effectiveness analysis of single and sequential testing strategies for tuberculosis infection in adults living with HIV in the United States. Scientific reports. 2022;12(1):18349.

S-112. G Gaetti, A Beneduce, D La Fauci, A Scardoni, F Chiappa, L Bellini, et al. Suture-Mediated Patent Foramen Ovale Closure Using the NobleStitch EL: Results from a Hospital-Based HTA. International journal of environmental research and public health. 2022;19(13).

S-113. G Li, YF Xia, YX Huang, D Okat, B Qiu, J Doyen, et al. Intensity-modulated proton radiation therapy as a radical treatment modality for nasopharyngeal carcinoma in China: Cost-effectiveness analysis. Head & neck. 2022;44(2):431-42.

S-114. G Li, YF Xia, YX Huang, D Okat, B Qiu, J Doyen, et al. Better preservation of erectile function in localized prostate cancer patients with modern proton therapy: Is it cost-effective? The Prostate. 2022;82(15):1438-46.

S-115. G Mourad, J Lundgren, G Andersson, M Husberg, P Johansson. Cost-effectiveness of internet-delivered cognitive behavioural therapy in patients with cardiovascular disease and depressive symptoms: secondary analysis of an RCT. BMJ open. 2022;12(4):e059939.

S-116. G Shao, J Wang, X Zhou, G Sun, Z Dong. Cost-effectiveness analysis of drug-eluting beads and conventional transarterial chemoembolization in the treatment of hepatocellular carcinoma. Frontiers in public health. 2022;10:963058.

S-117. G Shao, X Zhou, S Zhang, S Wu, Y Dong, Z Dong. Cost-utility analysis of centrally inserted totally implanted access port (PORT) vs. peripherally inserted central catheter (PICC) in the oncology chemotherapy. Frontiers in public health. 2022;10:942175.

S-118. GJ Chen, ME Kunik, CN Marti, NG Choi. Cost-effectiveness of Tele-delivered behavioral activation by Lay counselors for homebound older adults with depression. BMC psychiatry. 2022;22(1):648.

S-119. GJ Kirchner, NP Smith, ML Dunleavy, LE Nikkel. Intraoperative Imaging in Total Hip Arthroplasty Is Cost-Effective Regardless of Surgical Approach. The Journal of arthroplasty. 2022;37(8):S803-S6.

S-120. GL Botto, LG Mantovani, PA Cortesi, R De Ponti, A D'Onofrio, M Biffi, et al. The value of wearable cardioverter defibrillator in adult patients with recent myocardial infarction: Economic and clinical implications from a health technology assessment perspective. International journal of cardiology. 2022;356:12-8.

S-121. GMW Bjørnelv, Z Zolic-Karlsson, S Dueland, PD Line, E Aas. Cost-effectiveness of liver transplantation versus last-resort systemic therapy for colorectal liver metastases. The British journal of surgery. 2022;109(6):483-5.

S-122. H Albarmawi, KJ Cullen, R Mehra, E Onukwugha, O Goloubeva. Cost-effectiveness of concurrent radiation with cetuximab or chemotherapy in older patients with oropharyngeal cancer. Journal of comparative effectiveness research. 2022;11(8):595-607.

S-123. H Cutler, M Gumbie, E Olin, B Parkinson, R Bowman, H Quadri, T Mann. The cost-effectiveness of unilateral cochlear implants in UK adults. The European journal of health economics : HEPAC : health economics in prevention and care. 2022;23(5):763-79.

S-124. H Holmes, J McMaster, H Davies, V Vaines, J Turvill. Evaluation of the Cost-Utility of the York Faecal Calprotectin Care Pathway. Expert review of pharmacoeconomics & outcomes research. 2022;22(3):521-8.

S-125. H Ntuku, C Smith-Gueye, V Scott, J Njau, B Whittemore, B Zelman, et al. Cost and cost effectiveness of reactive case detection (RACD), reactive focal mass drug administration (rfMDA) and reactive focal vector control (RAVC) to reduce malaria in the low endemic setting of Namibia: an analysis alongside a 2×2 factorial design cluster randomised controlled trial. BMJ open. 2022;12(6):e049050.

S-126. H Sydow, S Prescher, F Koehler, K Koehler, M Dorenkamp, S Spethmann, et al. Cost-effectiveness of noninvasive telemedical interventional management in patients with heart failure: health economic analysis of the TIM-HF2 trial. Clinical research in cardiology : official journal of the German Cardiac Society. 2022;111(11):1231-44.

S-127. H Wang, C Huang, Y Yang, L Kong, X Zheng, X Shan. Cost-effectiveness analysis of nasojejunal tube feeding for the prevention of pneumonia in adults who are critically ill. JPEN Journal of parenteral and enteral nutrition. 2022;46(5):1167-75.

S-128. H Zheng, C Gong, R Chapman, L Yieh, P Friedlich, JW Hay. Cost-effectiveness analysis of extended extracorporeal membrane oxygenation duration in newborns with congenital diaphragmatic hernia in the United States. Pediatrics and neonatology. 2022;63(2):139-45.

S-129. H Zheng, GA Magee, TW Tan, DG Armstrong, WV Padula. Cost-effectiveness of Compression Therapy With Early Endovenous Ablation in Venous Ulceration for a Medicare Population. JAMA network open. 2022;5(12):e2248152.

S-130. HB Wolff, EMP Steeghs, ZA Mfumbilwa, HJM Groen, EM Adang, SM Willems, et al. Cost-Effectiveness of Parallel Versus Sequential Testing of Genetic Aberrations for Stage IV Non-Small-Cell Lung Cancer in the Netherlands. JCO precision oncology. 2022;6:e2200201.

S-131. HJ Chun, KN Cao, H Haruguchi, H Choi, M Yoshikawa, A Holden, JB Pietzsch. Economics of drug-coated balloons for arteriovenous fistula stenosis in Japan and Korea based on the IN.PACT AV access trial. Australia2022 2022-11. 859-68 p.

S-132. HR Graham, AA Bakare, AI Ayede, J Eleyinmi, O Olatunde, OR Bakare, et al. Cost-effectiveness and sustainability of improved hospital oxygen systems in Nigeria. BMJ global health. 2022;7(8).

S-133. HS Lim, S Shaw, AW Carter, S Jayawardana, E Mossialos, MR Mehra. A clinical and cost-effectiveness analysis of the HeartMate 3 left ventricular assist device for transplant-ineligible patients: A United Kingdom perspective. The Journal of heart and lung transplantation : the official publication of the International Society for Heart Transplantation. 2022;41(2):174-86.

S-134. HY Chan, BFM Wijnen, MHJM Majoie, SMAA Evers, M Hiligsmann. Economic evaluation of deep brain stimulation compared with vagus nerve stimulation and usual care for patients with refractory epilepsy: A lifetime decision analytic model. Epilepsia. 2022;63(3):641-51.

S-135. I Larrañaga, I Etxebarria-Foronda, O Ibarrondo, A Gorostiza, C Ojeda-Thies, JM Martínez-Llorente. Stratified cost-utility analysis of total hip arthroplasty in displaced femoral neck fracture. Gaceta sanitaria. 2022;36(1):12-8.

S-136. I Skarping, K Nilsson, L Dihge, A Fridhammar, M Ohlsson, L Huss, et al. The implementation of a noninvasive lymph node staging (NILS) preoperative prediction model is cost effective in primary breast cancer. Breast cancer research and treatment. 2022;194(3):577-86.

S-137. IGT Baeten, JP Hoogendam, GN Jonges, IM Jürgenliemk-Schulz, AJAT Braat, PJ van Diest, et al. Value of routine cytokeratin immunohistochemistry in detecting low volume disease in cervical cancer. Gynecologic oncology. 2022;165(2):257-63.

S-138. J Acolin. Economic Evaluation of Dialectical Behavioral Therapy Versus Cognitive Behavioral Therapy for Suicide Prevention. The journal of mental health policy and economics. 2022;25(4):123-31.

S-139. J Altunkaya, M Craven, S Lambe, A Beckley, L Rosebrock, R Dudley, et al. Estimating the Economic Value of Automated Virtual Reality Cognitive Therapy for Treating Agoraphobic Avoidance in Patients With Psychosis: Findings From the gameChange Randomized Controlled Clinical Trial. Journal of medical Internet research. 2022;24(11):e39248.

S-140. J Aaltio, V Hyttinen, M Kortelainen, GWJ Frederix, T Lönnqvist, A Suomalainen, P Isohanni. Cost-effectiveness of whole-exome sequencing in progressive neurological disorders of children. European journal of paediatric neurology : EJPN : official journal of the European Paediatric Neurology Society. 2022;36:30-6.

S-141. J Bjørdal, AN Fraser, TM Wagle, L Kleven, OA Lien, L Eilertsen, et al. A cost-effectiveness analysis of reverse total shoulder arthroplasty compared with locking plates in the management of displaced proximal humerus fractures in the elderly: the DelPhi trial. Journal of shoulder and elbow surgery. 2022;31(10):2187-95.

S-142. J Berdahl, C Bala, M Dhariwal, H Rathi, R Gupta. Cost-benefit analysis of a trifocal intraocular lens versus a monofocal intraocular lens from the patient's perspective in the United States. PloS one. 2022;17(11):e0277093.

S-143. J de Vos, LA Visser, AA de Beer, M Fornasa, PJ Thoral, PWG Elbers, G Cinà. The Potential Cost-Effectiveness of a Machine Learning Tool That Can Prevent Untimely Intensive Care Unit Discharge. Value in health : the journal of the International Society for Pharmacoeconomics and Outcomes Research. 2022;25(3):359-67.

S-144. J Ekersund, E Samuelsson, L Lindholm, M Sjöström. A mobile app for the treatment of female mixed and urgency incontinence: a cost-effectiveness analysis in Sweden. International urogynecology journal. 2022;33(5):1273-82.

S-145. J James, M Teo, V Ramachandran, M Law, M Cheng. Performance, clinical utility, and cost-effectiveness of selective use of staging investigations in early breast cancers. ANZ journal of surgery. 2022;92(3):426-30.

S-146. J Javan-Noughabi, A Rezapour, M Hajahmadi, V Alipour. Economic evaluation of single-photon emission-computed tomography versus stress echocardiography in stable chest pain patients. Scientific reports. 2022;12(1):15223.

S-147. J Karnon, HHA Afzali, B Bonevski. An Economic Evaluation of Government-Funded COVID-19 Testing in Australia. Applied health economics and health policy. 2022;20(5):681-91.

S-148. J Ory, F Bruyere, M Massetti, R Moreau. Economic evaluation of pre-operative shower with antiseptic soap to prevent surgical site infections. The Journal of hospital infection. 2022;124:9-12.

S-149. J Rosen, G Ceccon, EK Bauer, JM Werner, C Tscherpel, V Dunkl, et al. Cost Effectiveness of (18)F-FET PET for Early Treatment Response Assessment in Glioma Patients After Adjuvant Temozolomide Chemotherapy. Journal of nuclear medicine : official publication, Society of Nuclear Medicine. 2022;63(11):1677-82.

S-150. J Romsa, RJ Imhoff, SR Palli, R Inculet, S Mehta. SPECT/CT versus planar imaging to determine treatment strategy for non-small-cell lung cancer: a cost-effectiveness analysis. Journal of comparative effectiveness research. 2022;11(4):229-41.

S-151. J Singhirunnusorn, S Niyomsri, P Dilokthornsakul. The cost-effectiveness analysis of laparoscopic hepatectomy compared with open liver resection in the early stage of hepatocellular carcinoma: a decision-analysis model in Thailand. HPB : the official journal of the International Hepato Pancreato Biliary Association. 2022;24(2):183-91.

S-152. J Udkoff, BT Beal, DG Brodland, T Knackstedt. Cost effectiveness of intermediate-risk squamous cell carcinoma treated with Mohs micrographic surgery compared with wide local excision. Journal of the American Academy of Dermatology. 2022;86(2):303-11.

S-153. J van Santen, FJM Meiland, RM Dröes, A van Straten, JE Bosmans. Cost-effectiveness of exergaming compared to regular day-care activities in dementia: Results of a randomised controlled trial in The Netherlands. Health & social care in the community. 2022;30(5):e1794-e804.

S-154. J Wen, X Jin, F Al Sayah, JA Johnson, M Paulden, A Ohinmaa. Economic Evaluation of Sucrose Octasulfate Dressing for Treatment of Diabetic Foot Ulcers in Patients with Type 2 Diabetes. Canadian journal of diabetes. 2022;46(2):126-33.

S-155. J Yao, G Fekadu, X Jiang, JHS You. Telemonitoring for patients with inflammatory bowel disease amid the COVID-19 pandemic-A cost-effectiveness analysis. PloS one. 2022;17(4):e0266464.

S-156. J Yang, X Fan, J Gao, D Li, Y Xu, G Chen. Cost effectiveness analysis of total laparoscopic hysterectomy versus total abdominal hysterectomy for uterine fibroids in Western China: a societal perspective. BMC health services research. 2022;22(1):252.

S-157. JA Buendía, HL Talamoni. Cost-utility of use of sputum eosinophil counts to guide management in children with asthma. The Journal of asthma : official journal of the Association for the Care of Asthma. 2022;59(1):31-7.

S-158. JB Babigumira, CA Agutu, DT Hamilton, E van der Elst, A Hassan, E Gichuru, et al. Testing strategies to detect acute and prevalent HIV infection in adult outpatients seeking healthcare for symptoms compatible with acute HIV infection in Kenya: a cost-effectiveness analysis. BMJ open. 2022;12(9):e058636.

S-159. JB Bakkensen, KSJ Flannagan, SL Mumford, AP Hutchinson, EO Cheung, PI Moreno, et al. A SART data cost-effectiveness analysis of planned oocyte cryopreservation versus in vitro fertilization with preimplantation genetic testing for aneuploidy considering ideal family size. Fertility and sterility. 2022;118(5):875-84.

S-160. JC Prihadi, F Hafidz, H Djasri. Improvement of quality in clinical care for patients with benign prostatic hyperplasia: Cost effectiveness analysis. The Medical journal of Malaysia. 2022;77:10-5.

S-161. JE Aguilar-Nascimento, A Bicudo-Salomão, MRR Ribeiro, DB Dock-Nascimento, C Caporossi. COST-EFFECTIVENESS OF THE USE OF ACERTO PROTOCOL IN MAJOR DIGESTIVE SURGERY. Arquivos brasileiros de cirurgia digestiva : ABCD = Brazilian archives of digestive surgery. 2022;35:e1660.

S-162. JH Beard, ZM Thet Lwin, S Agarwal, M Ohene-Yeboah, S Tabiri, JKA Amoako, et al. Cost-Effectiveness Analysis of Inguinal Hernia Repair With Mesh Performed by Surgeons and Medical Doctors in Ghana. Value in health regional issues. 2022;32:31-8.

S-163. JHS You, SWC Luk, DYW Chow, X Jiang, ADP Mak, WWS Mak. Cost-effectiveness of internet-supported cognitive behavioral therapy for university students with anxiety symptoms: A Markov-model analysis. PloS one. 2022;17(5):e0268061.

S-164. JK Ngacha, R Ayah. Assessing the cost-effectiveness of contraceptive methods from a health provider perspective: case study of Kiambu County Hospital, Kenya. Reproductive health. 2022;19(1):11.

S-165. JK Peel, R Neves Miranda, D Naimark, G Woodward, MA Mamas, M Madan, HC Wijeysundera. Financial Incentives for Transcatheter Aortic Valve Implantation in Ontario, Canada: A Cost-Utility Analysis. Journal of the American Heart Association. 2022;11(8):e025085.

S-166. JM Boggs, DP Ritzwoller, A Beck, S Dimidjian, ZV Segal. Cost-Effectiveness of a Web-Based Program for Residual Depressive Symptoms: Mindful Mood Balance. Psychiatric services (Washington, DC). 2022;73(2):158-64.

S-167. JM Levin, J Wickman, AL Lazarides, DJ Cunningham, DE Goltz, RC Mather, et al. Is Advanced Imaging to Assess Rotator Cuff Integrity Before Shoulder Arthroplasty Cost-effective? A Decision Modeling Study. United States; 2022 2022-6-1. Report No.: 1528-1132 (Electronic) Contract No.: 6.

S-168. JP Ney, V Moll, EJ Kimball. Urinary catheter monitoring of intra-abdominal pressure after major abdominal surgery, a cost-benefit analysis. Journal of medical economics. 2022;25(1):412-20.

S-169. JP Sevilla, JM Klusty, Y Song, MJ Russo, CA Thompson, X Jiao, et al. Cost-utility and cost-benefit analysis of TAVR availability in the US severe symptomatic aortic stenosis patient population. Journal of medical economics. 2022;25(1):1051-60.

S-170. JR Acevedo, AC Hsu, JC Yu, DH Rice, DI Kwon, RW Kung, NC Kokot. Cost-effectiveness Analysis of Submandibular Gland Preservation With Sialendoscopy for the Management of Sialolithiasis. Otolaryngology--head and neck surgery : official journal of American Academy of Otolaryngology-Head and Neck Surgery. 2022;166(4):662-8.

S-171. JRM Colbourne, ST Alhayo, B Nandakumar, S Barat, W Liauwi, DL Morris, NA Alzahrani. Cost-effectiveness of Iterative Cytoreductive Surgery and Hyperthermic Intraperitoneal Chemotherapy for the Treatment of Peritoneal Carcinomatosis. In vivo (Athens, Greece). 2022;36(3):1527-33.

S-172. JS Rink, MF Froelich, JP McWilliams, C Gratzke, T Huber, E Gresser, et al. Prostatic Artery Emboliz ation for Treatment of Lower Urinary Tract Symptoms: A Markov Model-Based Cost-Effectiveness Analysis. Journal of the American College of Radiology : JACR. 2022;19(6):733-43.

S-173. JS Stone, AF Shaaban, WA Grobman, A Premkumar. Selective Fetoscopic Laser Photocoagulation or Expectant Management for Stage I Twin-Twin Transfusion: A Cost-Effectiveness Analysis. Fetal diagnosis and therapy. 2022;49(9):394-402.

S-174. K Donaldson, A Woll, SM Jansen, A Edenfield, S Swift, CA Heisler. A Cost-Effectiveness Analysis of Post-Void Residual Bladder Scan Thresholds in the Postoperative Setting. International urogynecology journal. 2022;33(10):2727-33.

S-175. K Dimitriadis, I Kyriopoulos, G Tsivgoulis, K Vemmos, WG Kunz, E Mossialos. Moving from traditional to more advanced treatments in stroke care is cost-effective: A case study from Greece. Journal of stroke and cerebrovascular diseases : the official journal of National Stroke Association. 2022;31(11):106764.

S-176. K Wang, Y Zhou, N Huang, Z Lu, X Zhang. Peripherally inserted central catheter versus totally implanted venous port for delivering medium- to long-term chemotherapy: A cost-effectiveness analysis based on propensity score matching. The journal of vascular access. 2022;23(3):365-74.

S-177. K Yun, J Yu, C Liu, X Zhang. A Cost-effectiveness Analysis of a Mobile Phone-Based Integrated HIV-Prevention Intervention Among Men Who Have Sex With Men in China: Economic Evaluation. Journal of medical Internet research. 2022;24(11):e38855.

S-178. K Yeung, W Zhu, SM McCurry, M Von Korff, R Wellman, CM Morin, MV Vitiello. Cost-effectiveness of telephone cognitive behavioral therapy for osteoarthritis-related insomnia. Journal of the American Geriatrics Society. 2022;70(1):188-99.

S-179. K Zhou, M Renouf, G Perrocheau, N Magné, I Latorzeff, P Pommier, et al. Cost-effectiveness of hypofractionated versus conventional radiotherapy in patients with intermediate-risk prostate cancer: An ancillary study of the PROstate fractionated irradiation trial - PROFIT. Radiotherapy and oncology : journal of the European Society for Therapeutic Radiology and Oncology. 2022;173:306-12.

S-180. KC Wall, GB Reddy, KL Corrigan, AP Toth, GE Garrigues. Operative Versus Nonoperative Management of Displaced Midshaft Clavicle Fractures: A Cost-effectiveness Analysis. Orthopedics. 2022;45(5):e243-e51.

S-181. KE Lee, F Lim, AS Faye, B Shen, C Hur. Endoscopic Balloon Dilation Is Cost-Effective for Crohn's Disease Strictures. Digestive diseases and sciences. 2022;67(12):5462-71.

S-182. KM Klifto, MG Tecce, JM Serletti, SJ Kovach. Comparison of nine methods of immediate breast reconstruction after resection of localized breast cancer: A cost-effectiveness Markov decision analysis of prospective studies. Microsurgery. 2022;42(5):401-27.

S-183. KR Chaudhuri, A Hand, F Obam, J Belsey. Cost-effectiveness analysis of the Parkinson's KinetiGraph and clinical assessment in the management of Parkinson's disease. Journal of medical economics. 2022;25(1):774-82.

S-184. L Caulley, E Krijkamp, MA Doyle, K Thavorn, F Alkherayf, N Sahlollbey, et al. Cost-effectiveness of direct surgery versus preoperative octreotide therapy for growth-hormone secreting pituitary adenomas. Pituitary. 2022;25(6):868-81.

S-185. L Claxton, M Walton, S Sharif-Hurst, R Wade, A Eastwood, R Hodgson. The Cost-Effectiveness of Selective Internal Radiation Therapies Compared With Sorafenib for Treating Advanced Unresectable Hepatocellular Carcinoma in the United Kingdom. Value in health : the journal of the International Society for Pharmacoeconomics and Outcomes Research. 2022;25(5):787-95.

S-186. L Gao, M Moodie, B Freedman, C Lam, H Tu, C Swift, et al. Cost-Effectiveness of Monitoring Patients Post-Stroke With Mobile ECG During the Hospital Stay. Journal of the American Heart Association. 2022;11(8):e022735.

S-187. L Manchikanti, R Kosanovic, V Pampati, MR Sanapati, A Soin, NN Knezevic, et al. Equivalent Outcomes of Lumbar Therapeutic Facet Joint Nerve Blocks and Radiofrequency Neurotomy: Comparative Evaluation of Clinical Outcomes and Cost Utility. Pain physician. 2022;25(2):179-92.

S-188. L Manchikanti, R Kosanovic, V Pampati, MR Sanapati, JA Hirsch. Outcomes of Cervical Therapeutic Medial Branch Blocks and Radiofrequency Neurotomy: Clinical Outcomes and Cost Utility are Equivalent. Pain physician. 2022;25(1):35-47.

S-189. L Mattia, S Davis, C Mark-Wagstaff, B Abrahamsen, N Peel, R Eastell, M Schini. Utility of PINP to monitor osteoporosis treatment in primary care, the POSE study (PINP and Osteoporosis in Sheffield Evaluation). Bone. 2022;158:116347.

S-190. L Verweij, ACM Petri, JL MacNeil-Vroomen, P Jepma, CHM Latour, RJG Peters, et al. The Cardiac Care Bridge transitional care program for the management of older high-risk cardiac patients: An economic evaluation alongside a randomized controlled trial. PloS one. 2022;17(1):e0263130.

S-191. LA Visser, M Folcher, C Delgado Simao, B Gutierrez Arechederra, E Escudero, CA Uyl-de Groot, WK Redekop. The Potential Cost-Effectiveness of a Cell-Based Bioelectronic Implantable Device Delivering Interferon-β1a Therapy Versus Injectable Interferon-β1a Treatment in Relapsing-Remitting Multiple Sclerosis. PharmacoEconomics. 2022;40(1):91-108.

S-192. LA van den Berg, OA Berkhemer, PSS Fransen, D Beumer, H Lingsma, CBM Majoie, et al. Economic Evaluation of Endovascular Treatment for Acute Ischemic Stroke. Stroke. 2022;53(3):968-75.

S-193. LE Barry, GE Crealey, P Cockwell, SJ Elliman, MD Griffin, AP Maxwell, et al. Mesenchymal stromal cell therapy compared to SGLT2-inhibitors and usual care in treating diabetic kidney disease: A cost-effectiveness analysis. United States2022 2022. e0274136 p.

S-194. LG Gordon, TM Elliott, C Bennett, G Hollway, N Waddell, L Vadlamudi. Early cost-utility analysis of genetically guided therapy for patients with drug-resistant epilepsy. Epilepsia. 2022;63(12):3111-21.

S-195. M Adee, H Zhong, EI Reipold, Y Zhuo, S Shilton, J Chhatwal. Cost-Effectiveness of a Core Antigen-Based Rapid Diagnostic Test for Hepatitis C. Value in health : the journal of the International Society for Pharmacoeconomics and Outcomes Research. 2022;25(7):1107-15.

S-196. M Ambrens, KS van Schooten, T Lung, L Clemson, JCT Close, K Howard, et al. Economic evaluation of the e-Health StandingTall balance exercise programme for fall prevention in people aged 70 years and over. Age and ageing. 2022;51(6).

S-197. M Assanatham, O Pattanaprateep, A Chuasuwan, K Vareesangthip, O Supasyndh, A Lumpaopong, et al. Economic evaluation of peritoneal dialysis and hemodialysis in Thai population with End-stage Kidney Disease. BMC health services research. 2022;22(1):1384.

S-198. M Comas, L Domingo, A Jansana, E Lafuente, A Civit, L García-Pérez, et al. Cost-effectiveness Analysis of Peripherally Inserted Central Catheters Versus Central Venous Catheters for in-Hospital Parenteral Nutrition. Journal of patient safety. 2022;18(7):e1109-e15.

S-199. M Caillon, R Sabatier, D Legallois, L Courouve, V Donio, F Boudevin, et al. A telemonitoring programme in patients with heart failure in France: a cost-utility analysis. BMC cardiovascular disorders. 2022;22(1):441.

S-200. M Dreyfuss, M Cohen-Vaizer, A Rokade, RD Laske. Cost-Effectiveness of Septorhinoplasty. United States; 2022 2022-5. Report No.: 2689-3622 (Electronic) Contract No.: 3.

S-201. M Dieng, RM Turner, SJ Lord, AJ Einstein, AM Menzies, RPM Saw, et al. Cost-Effectiveness of PET/CT Surveillance Schedules to Detect Distant Recurrence of Resected Stage III Melanoma. International journal of environmental research and public health. 2022;19(4).

S-202. M Gilard, H Eltchaninoff, B Iung, T Lefèvre, C Spaulding, N Dumonteil, et al. Cost-Effectiveness Analysis of SAPIEN 3 Transcatheter Aortic Valve Implantation Procedure Compared With Surgery in Patients With Severe Aortic Stenosis at Low Risk of Surgical Mortality in France. Value in health : the journal of the International Society for Pharmacoeconomics and Outcomes Research. 2022;25(4):605-13.

S-203. M Greuter, JJ Eertink, G Jongeneel, U Dührsen, A Hüttmann, C Schmitz, et al. Cost-Effectiveness of Shortening Treatment Duration Based on Interim PET Outcome in Patients With Diffuse Large B-cell Lymphoma. Clinical lymphoma, myeloma & leukemia. 2022;22(6):382-92.

S-204. M Howell, A Lawson, J Naylor, K Howard, IA Harris. Surgical plating versus closed reduction for fractures in the distal radius in older patients: a cost-effectiveness analysis from the hospital perspective. ANZ journal of surgery. 2022;92(12):3311-8.

S-205. M Javanbakht, A Mashayekhi, M Branagan-Harris, P Horvath, A Königsrainer, MA Reymond, M Yaghoubi. Cost-effectiveness analysis of pressurized intraperitoneal aerosol chemotherapy (PIPAC) in patients with gastric cancer and peritoneal metastasis. European journal of surgical oncology : the journal of the European Society of Surgical Oncology and the British Association of Surgical Oncology. 2022;48(1):188-96.

S-206. M Kiflen, A Le, S Mao, R Lali, S Narula, F Xie, G Paré. Cost-Effectiveness of Polygenic Risk Scores to Guide Statin Therapy for Cardiovascular Disease Prevention. Circulation Genomic and precision medicine. 2022;15(5):e003423.

S-207. M Kurnaz, S Ökçün, G Kahveci, S Şen, G Koçkaya. Cost-Effectiveness Analysis of the Triclip™ Transcatheter Tricuspid Valve Repair System in Patients with Tricuspid Regurgitation. Anatolian journal of cardiology. 2022;26(3):198-209.

S-208. M Khunte, X Wu, EW Avery, D Gandhi, S Payabvash, C Matouk, et al. Impact of collateral flow on cost-effectiveness of endovascular thrombectomy. Journal of neurosurgery. 2022;137(6):1801-10.

S-209. M Lindenberg, A Kramer, E Kok, V Retèl, G Beets, T Ruers, W van Harten. Image-guided navigation for locally advanced primary and locally recurrent rectal cancer: evaluation of its early cost-effectiveness. BMC cancer. 2022;22(1):504.

S-210. M Little, AM Gray, DG Altman, U Benedetto, M Flather, S Gerry, et al. Cost-effectiveness of bilateral vs. single internal thoracic artery grafts at 10 years. European heart journal Quality of care & clinical outcomes. 2022;8(3):324-32.

S-211. M Labban, P Dasgupta, C Song, R Becker, Y Li, US Kreaden, QD Trinh. Cost-effectiveness of Robotic-Assisted Radical Prostatectomy for Localized Prostate Cancer in the UK. JAMA network open. 2022;5(4):e225740.

S-212. M Mayo-Yáñez, C Chiesa-Estomba, JR Lechien, C Calvo-Henríquez, LA Vaira, I Cabo-Varela. Long-term outcomes and cost-effectiveness of a magnet-based valve voice prosthesis for endoprosthesis leakage treatment. European archives of oto-rhino-laryngology : official journal of the European Federation of Oto-Rhino-Laryngological Societies (EUFOS) : affiliated with the German Society for Oto-Rhino-Laryngology - Head and Neck Surgery. 2022;279(8):4167-72.

S-213. M Nord, J Lyth, J Marcusson, J Alwin. Cost-Effectiveness of Comprehensive Geriatric Assessment Adapted to Primary Care. Journal of the American Medical Directors Association. 2022;23(12):2003-9.

S-214. M Sekiguchi, A Igarashi, Y Mizuguchi, H Takamaru, M Yamada, T Sakamoto, et al. Cost-effectiveness analysis of endoscopic resection for colorectal laterally spreading tumors: Endoscopic submucosal dissection versus piecemeal endoscopic mucosal resection. Digestive endoscopy : official journal of the Japan Gastroenterological Endoscopy Society. 2022;34(3):553-68.

S-215. M Su, S Page, M Haag, K Swisshelm, D Hennerich, S Graw, et al. Clinical utility and cost-effectiveness analysis of chromosome testing concomitant with chromosomal microarray of patients with constitutional disorders in a U.S. academic medical center. Journal of genetic counseling. 2022;31(2):364-74.

S-216. M Yamamoto, H Yasunaga, R Kakinoki, N Tsubokawa, A Morita, K Tanaka, et al. The CeCORD-J study on collagenase injection versus aponeurectomy for Dupuytren's contracture compared by hand function and cost effectiveness. England2022 2022-5-31. 9094 p.

S-217. M Yoo, K Madaras-Kelly, M Nevers, KE Fleming-Dutra, AL Hersh, J Ying, et al. A Veterans' Healthcare Administration (VHA) antibiotic stewardship intervention to improve outpatient antibiotic use for acute respiratory infections: A cost-effectiveness analysis. Infection control and hospital epidemiology. 2022;43(10):1389-95.

S-218. MA Lindenberg, VP Retèl, HG van der Poel, F Bandstra, C Wijburg, WH van Harten. Cost-utility analysis on robot-assisted and laparoscopic prostatectomy based on long-term functional outcomes. Scientific reports. 2022;12(1):7658.

S-219. MA Rotondi, O Wong, M Riddell, B Perkins. Population-Level Impact and Cost-effectiveness of Continuous Glucose Monitoring and Intermittently Scanned Continuous Glucose Monitoring Technologies for Adults With Type 1 Diabetes in Canada: A Modeling Study. Diabetes care. 2022;45(9):2012-9.

S-220. MA van Kessel, CT Pham, R Tros, GJE Oosterhuis, WKH Kuchenbecker, MY Bongers, et al. The cost-effectiveness of transvaginal hydrolaparoscopy versus hysterosalpingography in the work-up for subfertility. Human reproduction (Oxford, England). 2022;37(12):2768-76.

S-221. MB Mitchell, RF Labadie. Cost-Effectiveness of Intraoperative CT Scanning in Cochlear Implantation in Fee-for-Service and Bundled Payment Models. Ear, nose, & throat journal. 2022;101(4):NP164-NP8.

S-222. MC Lee, CW Chang, SL Shih, SJ Huang, JY Tsauo, KL Hsiao, MY Chien. Efficacy and cost-effectiveness analysis of post-acute care for elderly patients with hip fractures. Journal of the Formosan Medical Association = Taiwan yi zhi. 2022;121(8):1596-604.

S-223. MD Jones, BD Franklin, DK Raynor, H Thom, MC Watson, R Kandiyali. Costs and Cost-Effectiveness of User-Testing of Health Professionals' Guidelines to Reduce the Frequency of Intravenous Medicines Administration Errors by Nurses in the United Kingdom: A Probabilistic Model Based on Voriconazole Administration. Applied health economics and health policy. 2022;20(1):91-104.

S-224. ME Moretti, J Jegathisawaran, G Wahi, A Bayliss, R Kanani, CM Pound, et al. Cost-effectiveness of Intermittent vs Continuous Pulse Oximetry Monitoring in Infants Hospitalized With Stabilized Bronchiolitis. JAMA network open. 2022;5(11):e2243609.

S-225. ME Png, S Petrou, J Achten, A Ooms, SE Lamb, H Hedley, et al. Cost-utility analysis of surgical fixation with Kirschner wire versus casting after fracture of the distal radius : a health economic evaluation of the DRAFFT2 trial. The bone & joint journal. 2022;104(11):1225-33.

S-226. ME Png, S Petrou, MA Fernandez, J Achten, N Parsons, A McGibbon, et al. Cost-utility analysis of cemented hemiarthroplasty versus hydroxyapatite-coated uncemented hemiarthroplasty for the treatment of displaced intracapsular hip fractures : the World Hip Trauma Evaluation 5 (WHiTE 5) trial. The bone & joint journal. 2022;104(8):922-8.

S-227. MJ Blackowicz, M Bell, J Echeverri, K Harenski, ME Broman. Cost-effectiveness of the TherMax blood warmer during continuous renal replacement therapy. PloS one. 2022;17(2):e0263054.

S-228. MJ Desai, A Bentley, WA Keck. Cooled radiofrequency ablation of the genicular nerves for chronic pain due to osteoarthritis of the knee: a cost-effectiveness analysis compared with intra-articular hyaluronan injections based on trial data. BMC musculoskeletal disorders. 2022;23(1):491.

S-229. MJ Steflik, BG Griswold, DV Patel, JA Blair, JM Davis. Antibiotic cement-coated intramedullary nail is cost-effective for the initial treatment of GAⅢ open tibia fractures. Injury. 2022;53(10):3471-4.

S-230. ML Chatterton, M Harris, P Burgess, S Fletcher, MJ Spittal, J Faller, et al. Economic evaluation of a Decision Support Tool to guide intensity of mental health care in general practice: the Link-me pragmatic randomised controlled trial. BMC primary care. 2022;23(1):236.

S-231. MM Hammer, CY Kong. Cost-Effectiveness of Follow-Up Ultrasound for Incidental Thyroid Nodules on CT. AJR American journal of roentgenology. 2022;218(4):615-22.

S-232. MM Salem, M Salih, F Nwajei, S Gomez-Paz, AJ Thomas, CS Ogilvy, JM Moore. Cost-Effectiveness Analytic Comparison of Neuroimaging Follow-Up Strategies After Pipeline Embolization Device Treatment of Unruptured Intracranial Aneurysms. World neurosurgery. 2022;158:e206-e13.

S-233. MP Subramanian, Z Yang, SH Chang, D Willis, J Zhang, TR Semenkovich, et al. Minimum Volume Standards for Surgical Care of Early-Stage Lung Cancer: A Cost-Effectiveness Analysis. The Annals of thoracic surgery. 2022;114(6):2001-7.

S-234. MS Lee, MA Assmus, DK Agarwal, ME Rivera, T Large, AE Krambeck. Ambulatory Percutaneous Nephrolithotomy May Be Cost-Effective Compared to Standard Percutaneous Nephrolithotomy. Journal of endourology. 2022;36(2):176-82.

S-235. MS Nazir, Y Rodriguez-Guadarrama, T Rua, KH Bui, A Buylova Gola, A Chiribiri, et al. Cost-effectiveness in diagnosis of stable angina patients: a decision-analytical modelling approach. Open heart. 2022;9(1).

S-236. MS Orlando, LA Cadish, JP Shepherd, T Falcone, OH Chang, RM Kho. Salpingo-oophorectomy or surveillance for ovarian endometrioma in asymptomatic premenopausal women: a cost-effectiveness analysis. American journal of obstetrics and gynecology. 2022;227(2):311.e1-.e7.

S-237. MT Liao, MH Lin, HE Tsai, JH Wu, JL Caffrey, JW Lin, et al. Risk stratification and cost-effectiveness analysis of adult patients receiving extracorporeal membrane oxygenation. Journal of evaluation in clinical practice. 2022;28(4):615-23.

S-238. MW Hnit, TM Han, L Nicodemus. Accuracy and Cost-effectiveness of the Diabetic Foot Screen Proforma in Detection of Diabetic Peripheral Neuropathy in Myanmar. Journal of the ASEAN Federation of Endocrine Societies. 2022;37(1):31-7.

S-239. MY Lin, CT Lee, MT Hsieh, MC Ou, YS Wang, MC Lee, et al. Endoscopic ultrasound avoids adverse events in high probability choledocholithiasis patients with a negative computed tomography. BMC gastroenterology. 2022;22(1):94.

S-240. N Bhattarai, CI Price, P McMeekin, M Javanbakht, L Vale, GA Ford, L Shaw. Cost-effectiveness of an enhanced Paramedic Acute Stroke Treatment Assessment (PASTA) during emergency stroke care: Economic results from a pragmatic cluster randomized trial. International journal of stroke : official journal of the International Stroke Society. 2022;17(3):282-90.

S-241. N Dharampal, K Smith, A Harvey, R Paschke, L Rudmik, S Chandarana. Cost-effectiveness analysis of molecular testing for cytologically indeterminate thyroid nodules. Journal of otolaryngology - head & neck surgery = Le Journal d'oto-rhino-laryngologie et de chirurgie cervico-faciale. 2022;51(1):46.

S-242. N Faccioli, E Santi, G Foti, G Mansueto, M Corain. Cost-effectiveness of introducing cone-beam computed tomography (CBCT) in the management of complex phalangeal fractures: economic simulation. Musculoskeletal surgery. 2022;106(2):169-77.

S-243. N Faccioli, E Santi, G Foti, M D'Onofrio. Cost-effectiveness analysis of including contrast-enhanced ultrasound in management of pancreatic cystic neoplasms. La Radiologia medica. 2022;127(4):349-59.

S-244. N Fujita, S Hatakeyama, M Momota, Y Tobisawa, T Yoneyama, H Yamamoto, et al. Risk score-based substratification improves surveillance costs after transurethral resection of bladder tumor in patients with primary high-risk non-muscle-invasive bladder cancer. Scientific reports. 2022;12(1):13786.

S-245. N Hezagira, S Youngkong, A Riewpaiboon. Cost-Utility Analysis of Community Case Management for Malaria Control in Burundi. International journal of health policy and management. 2022;11(12):2990-9.

S-246. N Omidifar, E Chogani, V Zangouri, K Keshavarz, A Talei. Cost-Effectiveness Analysis of Intraoperative Frozen Section in Women with Breast Cancer: Evidence from South of Iran. Iranian journal of medical sciences. 2022;47(2):143-51.

S-247. N Sivapragasam, DB Matchar, KD Zhuang, A Patel, U Pua, HH Win, et al. Cost-Effectiveness of Drug-Coated Balloon Angioplasty Versus Conventional Balloon Angioplasty for Treating Below-the-Knee Arteries in Chronic Limb-Threatening Ischemia: The SINGA-PACLI Trial. Cardiovascular and interventional radiology. 2022;45(11):1663-9.

S-248. N Schallner, J Lieberum, J Kalbhenn, H Bürkle, F Daumann. Intensive care unit resources and patient-centred outcomes in severe COVID-19: a prospective single-centre economic evaluation. Anaesthesia. 2022;77(12):1336-45.

S-249. N Wei, B Liu, M Ma, X Zhang, W Zhang, F Hou, et al. Patent foramen ovale closure vs. medical therapy alone after cryptogenic stroke in China: A cost-effectiveness analysis. Frontiers in public health. 2022;10:1016854.

S-250. ND Sridharan, RA Chaer, K Smith, MH Eslami. Carotid endarterectomy remains cost-effective for the surgical management of carotid stenosis. Journal of vascular surgery. 2022;75(4):1304-10.

S-251. NE van Hoogenhuijze, R van Eekelen, F Mol, I Schipper, ER Groenewoud, MAF Traas, et al. Economic evaluation of endometrial scratching before the second IVF/ICSI treatment: a cost-effectiveness analysis of a randomized controlled trial (SCRaTCH trial). Human reproduction (Oxford, England). 2022;37(2):254-63.

S-252. NR Gonzalez, MD Quintero-Consuegra, JL Chan, D Chang, CH Tseng, JL Saver. Cost-Effectiveness Analysis of Encephaloduroarteriosynangiosis Surgery for Symptomatic Intracranial Atherosclerotic Disease. Neurosurgery. 2022;90(4):495-500.

S-253. NR Pagani, MA Moverman, RN Puzzitiello, ME Menendez, JJ Kavolus. The Cost-Effectiveness of Closed Incisional Negative Pressure Wound Therapy for Infection Prevention after Revision Total Knee Arthroplasty. The journal of knee surgery. 2022;35(12):1301-5.

S-254. NS van Reijen, S van Dieren, FA Frans, JA Reekers, R Metz, HCJL Buscher, MJW Koelemay. Cost Effectiveness of Endovascular Revascularisation vs. Exercise Therapy for Intermittent Claudication Due to Iliac Artery Obstruction. European journal of vascular and endovascular surgery : the official journal of the European Society for Vascular Surgery. 2022;63(3):430-7.

S-255. OH Chang, JP Shepherd, B St Martin, ER Sokol, S Wallace. Surgical Correction of the Genital Hiatus at the Time of Sacrocolpopexy-Are Concurrent Posterior Repairs Cost-Effective? Female pelvic medicine & reconstructive surgery. 2022;28(5):325-31.

S-256. OH Chang, LA Cadish, A Kailasam, BM Ridgeway, JP Shepherd. Impact of the availability of midurethral slings on treatment strategies for stress urinary incontinence: a cost-effectiveness analysis. BJOG : an international journal of obstetrics and gynaecology. 2022;129(3):500-8.

S-257. OM Dong, PJ Poonnen, D Winski, SD Reed, V Vashistha, J Bates, et al. Cost-Effectiveness of Tumor Genomic Profiling to Guide First-Line Targeted Therapy Selection in Patients With Metastatic Lung Adenocarcinoma. Value in health : the journal of the International Society for Pharmacoeconomics and Outcomes Research. 2022;25(4):582-94.

S-258. P Andrén, M Holmsved, H Ringberg, V Wachtmeister, K Isomura, K Aspvall, et al. Therapist-Supported Internet-Delivered Exposure and Response Prevention for Children and Adolescents With Tourette Syndrome: A Randomized Clinical Trial. JAMA network open. 2022;5(8):e2225614.

S-259. P Duangthongphon, A Kitkhuandee, W Munkong, P Limwattananon, O Waleekhachonloet, T Rattanachotphanit, S Limwattananon. Cost-effectiveness analysis of endovascular coiling and neurosurgical clipping for aneurysmal subarachnoid hemorrhage in Thailand. Journal of neurointerventional surgery. 2022;14(9):942-7.

S-260. P Indraratna, U Biswas, J McVeigh, A Mamo, J Magdy, D Vickers, et al. A Smartphone-Based Model of Care to Support Patients With Cardiac Disease Transitioning From Hospital to the Community (TeleClinical Care): Pilot Randomized Controlled Trial. JMIR mHealth and uHealth. 2022;10(2):e32554.

S-261. P Lee, A Brennan, D Dinh, D Stub, J Lefkovits, CM Reid, et al. The cost-effectiveness of radial access percutaneous coronary intervention: A propensity-score matched analysis of Victorian data. Clinical cardiology. 2022;45(4):435-46.

S-262. P Lylyk, J Cirio, C Toranzo, E Aiello, J Valencia, D Paredes-Fernández. Mechanical Thrombectomy for Acute Ischemic Stroke Due to Large Vessel Occlusion in Argentina: An Economic Analysis. Journal of stroke and cerebrovascular diseases : the official journal of National Stroke Association. 2022;31(8):106595.

S-263. PB Lee, H Hojjat, J Lucas, MT Chung, A Spillinger, JB Meleca, et al. Cost-Effectiveness of Open vs. Endoscopic Repair of Zenker's Diverticulum. The Annals of otology, rhinology, and laryngology. 2022;131(5):499-505.

S-264. PG Carty, C Teljeur, CF De Gascun, P Gillespie, P Harrington, A McCormick, et al. Another Step Toward Hepatitis C Elimination: An Economic Evaluation of an Irish National Birth Cohort Testing Program. Value in health : the journal of the International Society for Pharmacoeconomics and Outcomes Research. 2022;25(12):1947-57.

S-265. PJ Belin, NA Yannuzzi, S Wagley, WE Smiddy, EH Ryan. COST ANALYSIS OF SCLERAL BUCKLE, PARS PLANA VITRECTOMY, AND PARS PLANA VITRECTOMY WITH SCLERAL BUCKLE FOR RETINAL DETACHMENT REPAIR. Retina (Philadelphia, Pa). 2022;42(1):33-7.

S-266. PJ Rohrbach, AE Dingemans, EF van Furth, P Spinhoven, JR van Ginkel, S Bauer, ME van den Akker-Van Marle. Cost-effectiveness of three internet-based interventions for eating disorders: A randomized controlled trial. The International journal of eating disorders. 2022;55(8):1143-55.

S-267. PL Quinn, S Bansal, A Gallagher, RJ Chokshi. Endoscopic Versus Laparoscopic Drainage of Pancreatic Pseudocysts: a Cost-effectiveness Analysis. Journal of gastrointestinal surgery : official journal of the Society for Surgery of the Alimentary Tract. 2022;26(8):1679-85.

S-268. PS Yesantharao, E Lee, KM Klifto, S Colakoglu, AL Dellon, SK Reddy. A Markov Analysis of Surgical versus Medical Management of Chronic Migraines. Plastic and reconstructive surgery. 2022;149(5):1187-96.

S-269. PV Rajan, A Khlopas, A Klika, R Molloy, V Krebs, NS Piuzzi. The Cost-Effectiveness of Robotic-Assisted Versus Manual Total Knee Arthroplasty: A Markov Model-Based Evaluation. The Journal of the American Academy of Orthopaedic Surgeons. 2022;30(4):168-76.

S-270. Q Zheng, L Shi, L Zhu, N Jiao, YS Chong, SW Chan, et al. Cost-effectiveness of Web-Based and Home-Based Postnatal Psychoeducational Interventions for First-time Mothers: Economic Evaluation Alongside Randomized Controlled Trial. Journal of medical Internet research. 2022;24(3):e25821.

S-271. R Caruso, E Vicente, Y Quijano, H Duran, E Diaz, I Fabra, et al. Case-matched analysis of robotic versus open surgical enucleation for pancreatic tumours: A comparative cost-effectiveness study. The international journal of medical robotics + computer assisted surgery : MRCAS. 2022;18(5):e2425.

S-272. R Diel, A Nienhaus. Point-of-care COVID-19 antigen testing in German emergency rooms - a cost-benefit analysis. Pulmonology. 2022;28(3):164-72.

S-273. R Hernandez, C Kennedy, K Banister, B Goulao, J Cook, S Sivaprasad, et al. Early detection of neovascular age-related macular degeneration: an economic evaluation based on data from the EDNA study. The British journal of ophthalmology. 2022;106(12):1754-61.

S-274. R Lewit, T Jancelewicz. Center Volume and Cost-Effectiveness in the Treatment of Congenital Diaphragmatic Hernia. The Journal of surgical research. 2022;273:71-8.

S-275. R Luengo-Fernandez, L Li, L Silver, S Gutnikov, NC Beddows, PM Rothwell. Long-Term Impact of Urgent Secondary Prevention After Transient Ischemic Attack and Minor Stroke: Ten-Year Follow-Up of the EXPRESS Study. Stroke. 2022;53(2):488-96.

S-276. R Quigley, N Verma, Jr Evuarherhe A, BJ Cole. Rotator Cuff Repair with Graft Augmentation Improves Function, Decreases Revisions, and Is Cost-Effective. Arthroscopy : the journal of arthroscopic & related surgery : official publication of the Arthroscopy Association of North America and the International Arthroscopy Association. 2022;38(7):2166-74.

S-277. R Song, V Jeet, R Sharma, M Hoyle, B Parkinson. Cost-Effectiveness Analysis of Prostate-Specific Membrane Antigen (PSMA) Positron Emission Tomography/Computed Tomography (PET/CT) for the Primary Staging of Prostate Cancer in Australia. PharmacoEconomics. 2022;40(8):807-21.

S-278. R Wang, P Tulikangas, HS Harvie. Apical Support Procedures at the Time of Hysterectomy for Benign Indications: A Cost-Effectiveness Analysis. Obstetrics and gynecology. 2022;139(5):788-96.

S-279. RA Glennie, JC Urquhart, P Koto, P Rasoulinejad, D Taylor, K Sequeira, et al. Microdiscectomy Is More Cost-effective Than a 6-Month Nonsurgical Care Regimen for Chronic Radiculopathy. United States2022 2022-3-1. 574-84 p.

S-280. RA Lourenco, T Khoo, A Crothers, M Haas, R Montgomery, D Ball, et al. Cost-Effectiveness of Single Versus Multifraction SABR for Pulmonary Oligometastases: The SAFRON II Trial. International journal of radiation oncology, biology, physics. 2022;114(5):968-76.

S-281. RB Mailhot Vega, H Mohammadi, SI Patel, AL Holtzman, NA Lockney, JW Lynch, et al. Establishing Cost-Effective Allocation of Proton Therapy for Patients With Mediastinal Hodgkin Lymphoma. International journal of radiation oncology, biology, physics. 2022;112(1):158-66.

S-282. RM Modi, CL Liu, N Isaza, I Raber, P Calvachi, P Zimetbaum, et al. Cost-Effectiveness of Antibiotic-Eluting Envelope for Prevention of Cardiac Implantable Electronic Device Infections in Heart Failure. Circulation Cardiovascular quality and outcomes. 2022;15(3):e008443.

S-283. RO Kowalchuk, TC Mullikin, DK Kim, JM Morris, DK Ebner, WS Harmsen, et al. Cost-Effectiveness of Treatment Strategies for Spinal Metastases. Practical radiation oncology. 2022;12(3):236-44.

S-284. RR Thompson, A Kityamuwesi, A Kuan, D Oyuku, A Tucker, O Ferguson, et al. Cost and Cost-Effectiveness of a Digital Adherence Technology for Tuberculosis Treatment Support in Uganda. Value in health : the journal of the International Society for Pharmacoeconomics and Outcomes Research. 2022;25(6):924-30.

S-285. RS See-Toh, XY Wong, KSKH Mahboobani, SS Soon, B Kearns, K Cooper, et al. Cost-effectiveness of transcatheter aortic valve implantation in patients with severe symptomatic aortic stenosis of intermediate surgical risk in Singapore. BMC health services research. 2022;22(1):994.

S-286. RW Treskes, ME van den Akker-van Marle, L van Winden, N van Keulen, ET van der Velde, S Beeres, et al. The Box-eHealth in the Outpatient Clinic Follow-up of Patients With Acute Myocardial Infarction: Cost-Utility Analysis. Journal of medical Internet research. 2022;24(4):e30236.

S-287. S Arjani, TJ Bostonian, V Prasath, PL Quinn, RJ Chokshi. Cost-effectiveness of adrenal vein sampling- vs computed tomography-guided adrenalectomy for unilateral adrenaloma in primary aldosteronism. Journal of endocrinological investigation. 2022;45(10):1899-908.

S-288. S Du, J Zhao, G Qiao, S Wu, Y Han. Cost-Effectiveness Analysis of the Application of a Porcine-Derived Fibrin Sealant for the Treatment of Cerebrospinal Fluid Leak in China. Clinical therapeutics. 2022;44(4):575-84.

S-289. S Dioun, L Chen, A Melamed, A Gockley, CM St Clair, JY Hou, et al. Minimally invasive surgery for suspected early-stage ovarian cancer; a cost-effectiveness study. BJOG : an international journal of obstetrics and gynaecology. 2022;129(5):777-84.

S-290. S Eluri, A Paterson, BN Lauren, M O'Donovan, P Bhandari, M di Pietro, et al. Utility and Cost-Effectiveness of a Nonendoscopic Approach to Barrett's Esophagus Surveillance After Endoscopic Therapy. Clinical gastroenterology and hepatology : the official clinical practice journal of the American Gastroenterological Association. 2022;20(2):e51-e63.

S-291. S Ellmann, M Maryschok, O Schöffski, M Emmert. The German COVID-19 Digital Contact Tracing App: A Socioeconomic Evaluation. International journal of environmental research and public health. 2022;19(21).

S-292. S Fuentes, J Núñez-Alfonsel, JM Pradillos-Serna, C Grande-Moreillo, J Margarit-Mallol, S Valladares-Díez, E Ardela-Díaz. Quality of Life in Pediatric Minimally Invasive Surgery: Cost-Utility Analysis of Laparoscopic Versus Open Appendectomy. Journal of laparoendoscopic & advanced surgical techniques Part A. 2022;32(2):219-25.

S-293. S Gyftopoulos, J Conroy, J Koo, M Jones, A Miniaci, N Subhas. Imaging of Patients Suspected of SLAP Tear: A Cost-Effectiveness Study. AJR American journal of roentgenology. 2022;218(2):227-33.

S-294. S Jiang, PC Mathias, N Hendrix, BH Shirts, P Tarczy-Hornoch, D Veenstra, et al. Implementation of pharmacogenomic clinical decision support for health systems: a cost-utility analysis. The pharmacogenomics journal. 2022;22(3):188-97.

S-295. S Kepka, K Zarca, D Viglino, N Marjanovic, O Taheri, O Peyrony, et al. Imaging strategies used in emergency departments for the diagnostic workup of COVID-19 patients during the first wave of the pandemic: a cost-effectiveness analysis. Clinical microbiology and infection : the official publication of the European Society of Clinical Microbiology and Infectious Diseases. 2022;28(12):1651.e1-.e8.

S-296. S Kepka, K Zarca, F Lersy, M Moris, J Godet, J Deur, et al. MRI dedicated to the emergency department for diplopia or dizziness: a cost-effectiveness analysis. European radiology. 2022;32(11):7344-53.

S-297. S Liu, Y Lv, M Liu, S Han, X Liu, Z Zhao, et al. Luteinizing hormone-based modified GnRH antagonist protocol in normal responders undergoing in vitro fertilization treatment: A multi-center randomized controlled trial. Frontiers in endocrinology. 2022;13:922950.

S-298. S Okazaki, K Shibuya, T Takura, Y Miyasaka, H Kawamura, T Ohno. Cost-effectiveness of carbon-ion radiotherapy versus stereotactic body radiotherapy for non-small-cell lung cancer. England2022 2022-2. 674-83 p.

S-299. S Putri, RR Nugraha, E Pujiyanti, H Thabrany, H Hasnur, ND Istanti, et al. Supporting dialysis policy for end stage renal disease (ESRD) in Indonesia: an updated cost-effectiveness model. BMC research notes. 2022;15(1):359.

S-300. S Paganini, Y Terhorst, LB Sander, J Lin, S Schlicker, DD Ebert, et al. Internet- and mobile-based intervention for depression in adults with chronic back pain: A health economic evaluation. Journal of affective disorders. 2022;308:607-15.

S-301. S Ranno, GM Rabbiolo, S Lucentini, E Ruggiero, SV Luccarelli, L Lombardi, P Nucci. Angle-supported intraocular lens versus scleral-sutured posterior chamber intraocular lens in post-cataract surgery aphakic patients: two-year follow-up cost-effectiveness analysis. International ophthalmology. 2022;42(3):871-9.

S-302. S Sood, N Heilenbach, V Sanchez, S Glied, S Chen, LA Al-Aswad. Cost-Effectiveness Analysis of Minimally Invasive Trabecular Meshwork Stents with Phacoemulsification. Ophthalmology Glaucoma. 2022;5(3):284-96.

S-303. S Singh, S Hearps, DK Nishijima, JA Cheek, M Borland, S Dalziel, et al. Cost-effectiveness of patient observation on cranial CT use with minor head trauma. Archives of disease in childhood. 2022;107(8):712-8.

S-304. S Stéphane, W Timothée, A Jérémie, O Raphael, D Martin, P Emmanuelle, et al. Endoscopic submucosal dissection or piecemeal endoscopic mucosal resection for large superficial colorectal lesions: A cost effectiveness study. Clinics and research in hepatology and gastroenterology. 2022;46(6):101969.

S-305. S Techapongsatorn, A Tansawet, O Pattanaprateep, J Attia, GJ Mckay, A Thakkinstian. Cost-effectiveness analysis of mesh fixation techniques for laparoscopic and open inguinal hernia surgeries. BMC health services research. 2022;22(1):1125.

S-306. S Vicente-Jiménez, B Lopez-Valcarcel, M Maynar, E Perez-Fernández, P Carrasco, G Rodriguez-Caravaca, et al. Clinical results and cost-effectiveness of radiofrequency and cyanoacrylate ablation compared with traditional surgical stripping for treating varicose veins. Journal of vascular surgery Venous and lymphatic disorders. 2022;10(4):846-54.e2.

S-307. SC Resch, JHA Foote, KE Wirth, A Lasry, JA Scott, J Moore, et al. Health Impact and Cost-Effectiveness of HIV Testing, Linkage, and Early Antiretroviral Treatment in the Botswana Combination Prevention Project. Journal of acquired immune deficiency syndromes (1999). 2022;90(4):399-407.

S-308. SG Vitale, AS Laganà, P Török, RB Lasmar, J Carugno, M Palumbo, J Tesarik. Virtual sonographic hysteroscopy in assisted reproduction: A retrospective cost-effectiveness analysis. International journal of gynaecology and obstetrics: the official organ of the International Federation of Gynaecology and Obstetrics. 2022;156(1):112-8.

S-309. SM McPhail, J Wiseman, M Simons, R Kimble, Z Tyack. Cost-effectiveness of scar management post-burn: a trial-based economic evaluation of three intervention models. Scientific reports. 2022;12(1):18601.

S-310. SN Frempong, B Shinkins, D Howdon, M Messenger, RD Neal, GS Sagoo. Early economic evaluation of an intervention to improve uptake of the NHS England Diabetes Prevention Programme. Expert review of pharmacoeconomics & outcomes research. 2022;22(3):417-27.

S-311. SR Chaiken, JA Bohn, AS Bruegl, AB Caughey, EG Munro. Hysterectomy with a general gynecologist vs gynecologic-oncologist in the setting of endometrial intraepithelial neoplasia: a cost-effectiveness analysis. American journal of obstetrics and gynecology. 2022;227(4):609.e1-.e8.

S-312. SRW Wijn, MA Hentschel, AJ Beynon, HPM Kunst, MM Rovers. Auditory brainstem response prior to MRI compared to standalone MRI in the detection of vestibular schwannoma: A modelling study. England2022 2022-3. 295-303 p.

S-313. ST Gregory, WK Goodman, B Kay, B Riemann, EA Storch. Cost-effectiveness analysis of deep transcranial magnetic stimulation relative to evidence-based strategies for treatment-refractory obsessive-compulsive disorder. Journal of psychiatric research. 2022;146:50-4.

S-314. T Begisbayev, L Kosherbayeva, K Gaitova, M Brimzhanova. Cost-Effectiveness of Cardioverter-Defibrillator Implantation in Kazakhstan. Vascular health and risk management. 2022;18:813-21.

S-315. T Mou, LA Cadish, EL Gray, CE Bretschneider. Cost-effectiveness of prophylactic retropubic sling at the time of vaginal prolapse surgery. American journal of obstetrics and gynecology. 2022;227(3):471.e1-.e7.

S-316. T Noda, X Lu, Y Ishiguro, J Ikuemonisan, R Holbrook, S Tsintzos, K Kusano. Cost-effective analysis of automated programming optimization in cardiac resynchronization therapy: Holistic Markov modelling. Journal of cardiology. 2022;79(6):734-9.

S-317. T Pelle, K Bevers, F van den Hoogen, J van der Palen, C van den Ende. Economic Evaluation of the Dr. Bart Application in Individuals With Knee and/or Hip Osteoarthritis. Arthritis care & research. 2022;74(6):945-54.

S-318. T Shiina, K Goto-Hirano, T Takura, H Daida. Cost-effectiveness of follow-up invasive coronary angiography after percutaneous coronary stenting: a real-world observational cohort study in Japan. BMJ open. 2022;12(8):e061617.

S-319. T Sprave, V Verma, A Fabian, A Rühle, D Baltas, AL Grosu, NH Nicolay. Cost effectiveness and health-related quality of life of chemoradiotherapy versus radiation therapy alone in elderly head and neck cancer patients. Strahlentherapie und Onkologie : Organ der Deutschen Rontgengesellschaft [et al]. 2022;198(11):1008-15.

S-320. T Takura, H Yokoi, N Tanaka, N Matsumoto, E Yoshida, T Nakata. Health economics-based verification of functional myocardial ischemia evaluation of stable coronary artery disease in Japan: A long-term longitudinal study using propensity score matching. Journal of nuclear cardiology : official publication of the American Society of Nuclear Cardiology. 2022;29(3):1356-69.

S-321. TA Crittenden, J Ratcliffe, DI Watson, C Mpundu-Kaambwa, NR Dean. Cost-utility analysis of breast reduction surgery for women with symptomatic breast hypertrophy. The Medical journal of Australia. 2022;216(3):147-52.

S-322. TA Kanters, CPAM Raaijmakers, PNM Lohle, J de Vries, L Hakkaart-van Roijen. Cost Effectiveness of Splenic Artery Embolization versus Splenectomy after Trauma in the Netherlands. Journal of vascular and interventional radiology : JVIR. 2022;33(4):392-8.e4.

S-323. TA Lavelle, X Feng, M Keisler, JT Cohen, PJ Neumann, D Prichard, et al. Cost-effectiveness of exome and genome sequencing for children with rare and undiagnosed conditions. Genetics in medicine : official journal of the American College of Medical Genetics. 2022;24(6):1349-61.

S-324. TJ Abel, M Remick, WC Welch, KJ Smith. One-year cost-effectiveness of callosotomy vs vagus nerve stimulation for drug-resistant seizures in Lennox-Gastaut Syndrome: A decision analytic model. Epilepsia open. 2022;7(1):124-30.

S-325. TJ Orellana, H Kim, S Beriwal, R Bhargava, J Berger, RJ Buckanovich, et al. Cost-effectiveness analysis of tumor molecular classification in high-risk early-stage endometrial cancer. Gynecologic oncology. 2022;164(1):129-35.

S-326. TM Boere, M El Alili, LW van Buul, RM Hopstaken, TJM Verheij, CMPM Hertogh, et al. Cost-effectiveness and return-on-investment of C-reactive protein point-of-care testing in comparison with usual care to reduce antibiotic prescribing for lower respiratory tract infections in nursing homes: a cluster randomised trial. BMJ open. 2022;12(9):e055234.

S-327. TR Johannessen, S Halvorsen, D Atar, J Munkhaugen, AK Nore, T Wisløff, OM Vallersnes. Cost-effectiveness of a rule-out algorithm of acute myocardial infarction in low-risk patients: emergency primary care versus hospital setting. BMC health services research. 2022;22(1):1274.

S-328. TSM de Assis, ML Freire, JP Carvalho, A Rabello, G Cota. Cost-effectiveness of anti-SARS-CoV-2 antibody diagnostic tests in Brazil. PloS one. 2022;17(2):e0264159.

S-329. TY Cras, MMG Hunink, R Dammers, ACGM van Es, V Volovici, JF Burke, et al. Surveillance of Unruptured Intracranial Aneurysms: Cost-Effectiveness Analysis for 3 Countries. Neurology. 2022;99(9):e890-e903.

S-330. UV Mahajan, DI Ojukwu, DE Azagury, DL Safer, T Cunningham, CH Halpern. Can responsive deep brain stimulation be a cost-effective treatment for severe obesity? Obesity (Silver Spring, Md). 2022;30(2):338-46.

S-331. V Berdunov, S Millen, A Paramore, P Hall, T Perren, R Brown, et al. Cost-effectiveness analysis of the Oncotype DX Breast Recurrence Score test in node-positive early breast cancer. Journal of medical economics. 2022;25(1):591-604.

S-332. V Eggerding, M Reijman, DE Meuffels, E van Es, E van Arkel, I van den Brand, et al. ACL reconstruction for all is not cost-effective after acute ACL rupture. British journal of sports medicine. 2022;56(1):24-8.

S-333. V Lambadiari, AZ Ozdemir Saltik, S de Portu, MI Buompensiere, A Kountouri, E Korakas, et al. Cost-Effectiveness Analysis of an Advanced Hybrid Closed-Loop Insulin Delivery System in People with Type 1 Diabetes in Greece. Diabetes technology & therapeutics. 2022;24(5):316-23.

S-334. V Prasath, PL Quinn, JB Oliver, S Arjani, SK Ahlawat, RJ Chokshi. Cost-effectiveness analysis of infected necrotizing pancreatitis management in an academic setting. Pancreatology : official journal of the International Association of Pancreatology (IAP) [et al]. 2022;22(2):185-93.

S-335. VM Veličković, M Szilcz, Z Milošević, T Godfrey, U Siebert. Cost-effectiveness analysis of superabsorbent wound dressings in patients with moderate-to-highly exuding leg ulcers in Germany. International wound journal. 2022;19(2):447-59.

S-336. VM Veličković, PA Prieto, M Krga, AM Jorge. Superabsorbent wound dressings versus foams dressings for the management of moderate-to-highly exuding venous leg ulcers in French settings: An early stage model-based economic evaluation. Journal of tissue viability. 2022;31(3):523-30.

S-337. VT Ho, AT Nguyen, JR Stern, SM Asch, DK Owens, JA Salomon, et al. Cost-effectiveness of computed tomography versus ultrasound-based surveillance following endovascular aortic repair of intact infrarenal abdominal aortic aneurysms. Journal of vascular surgery. 2022;76(3):707-13.e1.

S-338. W Rattanavipapong, T Worakijthamrongchai, B Soboon, V Luankongsomchit, M Kongmuangpuk, W Isaranuwatchai, et al. Economic evaluation of endovascular treatment for acute ischaemic stroke in Thailand. BMJ open. 2022;12(9):e064403.

S-339. WA Gray, RI Griffiths, PWM Elroy, SL Amorosi, AM McGovern, MR Jaff, et al. Cost-effectiveness of a paclitaxel-eluting stent (Eluvia) compared to Zilver PTX for endovascular femoropopliteal intervention. Journal of medical economics. 2022;25(1):880-7.

S-340. WH Tettelbach, DG Armstrong, TJ Chang, JL Jong, PM Glat, JH Hsu, et al. Cost-effectiveness of dehydrated human amnion/chorion membrane allografts in lower extremity diabetic ulcer treatment. Journal of wound care. 2022;31:S10-S31.

S-341. WJA Witlox, BLT Ramaekers, B Lacas, C Le Pechoux, A Sun, SY Wang, et al. Cost-effectiveness of prophylactic cranial irradiation in stage III non-small cell lung cancer. Ireland2022 2022-5. 95-101 p.

S-342. WV Padula, MA Miano, MA Kelley, SA Crawford, BH Choy, RM Hughes, et al. A Cost-Utility Analysis of Remote Pulse-Oximetry Monitoring of Patients With COVID-19. Value in health : the journal of the International Society for Pharmacoeconomics and Outcomes Research. 2022;25(6):890-6.

S-343. X Gao, YW Wen, JJB van Lanschot, YK Chao. Neoadjuvant Therapy Versus Upfront Surgery for Patients With Clinical Stage 2 or 3 Esophageal Squamous Cell Carcinoma: A Cost-Effectiveness Analysis. Annals of surgical oncology. 2022;29(6):3644-53.

S-344. X Luo, W Xu, WK Ming, X Jiang, Q Yuan, H Lai, et al. Cost-Effectiveness of Mobile Health-Based Integrated Care for Atrial Fibrillation: Model Development and Data Analysis. Journal of medical Internet research. 2022;24(4):e29408.

S-345. X Wu, A Zhou, M Heller, R Kohlbrenner. Prostatic Artery Embolization Versus Transurethral Resection of the Prostate for Benign Prostatic Hyperplasia: A Cost-Effectiveness Analysis. Journal of vascular and interventional radiology : JVIR. 2022;33(12):1605-15.

S-346. X Wu, EJ Keller, R Rabei, H Rockwell, S Beeson, M Heller, N Kothary. Cost-effectiveness of tunneled peritoneal catheters versus repeat paracenteses for recurrent ascites in gynecologic malignancies. Gynecologic oncology. 2022;164(3):639-44.

S-347. X Wu, J Uhlig, JD Blasberg, SN Gettinger, RD Suh, SB Solomon, HS Kim. Microwave Ablation versus Stereotactic Body Radiotherapy for Stage I Non-Small Cell Lung Cancer: A Cost-Effectiveness Analysis. Journal of vascular and interventional radiology : JVIR. 2022;33(8):964-71.e2.

S-348. X Weng, L Zhong, P Xiang, Y Li, A Paciorek, L Dong, et al. Cost-effectiveness analysis of primary treatments for localised prostate cancer: A population-based Markov analysis using real-world evidence. European journal of cancer care. 2022;31(6):e13740.

S-349. X Wu, R Rabei, EJ Keller, B King, N Kothary, M Kohi, et al. Tunneled Peritoneal Catheter vs Repeated Paracenteses for Recurrent Ascites: A Cost-Effectiveness Analysis. Cardiovascular and interventional radiology. 2022;45(7):972-82.

S-350. X Wang, Y Cai, B Zhang, X Zhang, L Wang, X Yan, et al. Cost-effectiveness analysis on COVID-19 surveillance strategy of large-scale sports competition. Infectious diseases of poverty. 2022;11(1):32.

S-351. XL Jiao, SC Li, L Hao, TG Wang, JF Chen. Cost-benefit analysis of hepatic resection, radiofrequency ablation and liver transplantation in small hepatocellular carcinoma. Expert review of pharmacoeconomics & outcomes research. 2022;22(2):307-13.

S-352. Y Feng, C Roukas, M Russo, S Repišti, A Džubur Kulenović, L Injac Stevović, et al. Cost-effectiveness of implementing a digital psychosocial intervention for patients with psychotic spectrum disorders in low- and middle-income countries in Southeast Europe: Economic evaluation alongside a cluster randomised trial. European psychiatry : the journal of the Association of European Psychiatrists. 2022;65(1):e56.

S-353. Y Ge, A Pandya, SAM Cuddy, A Singh, A Singh, S Dorbala. Modeling the Cost and Health Impacts of Diagnostic Strategies in Patients with Suspected Transthyretin Cardiac Amyloidosis. Journal of the American Heart Association. 2022;11(18):e026308.

S-354. Y Hua, J Salcedo. Cost-effectiveness analysis of robotic-arm assisted total knee arthroplasty. PloS one. 2022;17(11):e0277980.

S-355. Y Mares-Gutiérrez, G Salinas-Escudero, B Aracena-Genao, A Martínez-González, M García-Minjares, YN Flores. Preoperative risk assessment and spirometry is a cost-effective strategy to reduce post-operative complications and mortality in Mexico. PloS one. 2022;17(7):e0271953.

S-356. Y Shi, P Zhu, J Jia, Z Shao, S Yang, W Chen, et al. Cost-effectiveness of Same-day Discharge Surgery for Primary Total Hip Arthroplasty: A Pragmatic Randomized Controlled Study. Frontiers in public health. 2022;10:825727.

S-357. Y Shijoh, S Saito, Z Dai, S Ohde. Cost-effectiveness analysis of patent foramen ovale closure versus medical therapy alone after cryptogenic stroke. PloS one. 2022;17(6):e0268690.

S-358. YI Won, CH Kim, HP Park, SG Chung, WT Yuh, SW Kwon, et al. A cost-utility analysis between decompression only and fusion surgery for elderly patients with lumbar spinal stenosis and sagittal imbalance. Scientific reports. 2022;12(1):20408.

S-359. YJ Kang, M Caruana, K McLoughlin, J Killen, K Simms, N Taylor, et al. The predicted effect and cost-effectiveness of tailoring colonoscopic surveillance according to mismatch repair gene in patients with Lynch syndrome. Genetics in medicine : official journal of the American College of Medical Genetics. 2022;24(9):1831-46.

S-360. YN Aung, STT Tun, V Vanisaveth, K Chindavongsa, L Kanya. Cost-effectiveness analysis of G6PD diagnostic test for Plasmodium vivax radical cure in Lao PDR: An economic modelling study. PloS one. 2022;17(4):e0267193.

S-361. Z Ghani, S Saha, J Jarl, M Andersson, JS Berglund, P Anderberg. Short Term Economic Evaluation of the Digital Platform "Support, Monitoring and Reminder Technology for Mild Dementia" (SMART4MD) for People with Mild Cognitive Impairment and their Informal Caregivers. Journal of Alzheimer's disease : JAD. 2022;86(4):1629-41.

S-362. Z Zhang, Y Bao, L Cai, Y Gu, T Yang, X Li. Cost-Utility Analysis of CYP2C19 Genotype Detection for Selection of Acid-Suppressive Therapy with Lansoprazole or Vonoprazan for Patients with Reflux Esophagitis in China. Clinical drug investigation. 2022;42(10):839-51.

S-363. ZC Yang, ZQ Nie, QY Chen, CC Du, DH Luo, LT Liu, et al. Cost-Effectiveness analysis of combining plasma Epstein-Barr virus DNA testing and different surveillance imaging modalities for nasopharyngeal carcinoma patients in first remission. Oral oncology. 2022;128:105851.

S-364. ZZ Huang, R Mao, YQ Li, HZ Tian, M Iacucci, B Shen, et al. Cost-efficient snare-assisted peroral endoscopic myotomy in comparison of conventional endoscopic knife for treatment of achalasia: results of a randomized controlled trial. Diseases of the esophagus : official journal of the International Society for Diseases of the Esophagus. 2022;35(8).
